# Auditory sequence processing reveals evolutionarily conserved regions of frontal cortex in macaques and humans

**DOI:** 10.1038/ncomms9901

**Published:** 2015-11-17

**Authors:** Benjamin Wilson, Yukiko Kikuchi, Li Sun, David Hunter, Frederic Dick, Kenny Smith, Alexander Thiele, Timothy D. Griffiths, William D. Marslen-Wilson, Christopher I. Petkov

**Affiliations:** 1 Institute of Neuroscience, Henry Wellcome Building, Newcastle University, Framlington Place, Newcastle upon Tyne, NE2 4HH, UK; 2 Centre for Behaviour and Evolution, Henry Wellcome Building, Newcastle University, Framlington Place, Newcastle upon Tyne, NE2 4HH, UK; 3 Department of Psychological Sciences, Birkbeck University of London, London, WC1E 7HX, UK; 4 School of Philosophy, Psychology and Language Sciences, University of Edinburgh, Edinburgh, EH8 9AD, UK; 5 Department of Psychology, University of Cambridge, Cambridge, CB2 3EB, UK

## Abstract

An evolutionary account of human language as a neurobiological system must distinguish between human-unique neurocognitive processes supporting language and evolutionarily conserved, domain-general processes that can be traced back to our primate ancestors. Neuroimaging studies across species may determine whether candidate neural processes are supported by homologous, functionally conserved brain areas or by different neurobiological substrates. Here we use functional magnetic resonance imaging in *Rhesus macaques* and humans to examine the brain regions involved in processing the ordering relationships between auditory nonsense words in rule-based sequences. We find that key regions in the human ventral frontal and opercular cortex have functional counterparts in the monkey brain. These regions are also known to be associated with initial stages of human syntactic processing. This study raises the possibility that certain ventral frontal neural systems, which play a significant role in language function in modern humans, originally evolved to support domain-general abilities involved in sequence processing.

## Abstract (web summary)

This study uses functional magnetic resonance imaging in humans and monkeys to show similar ventral frontal and opercular cortical responses when processing sequences of auditory nonsense words. The study indicates that this frontal region is involved in evaluating the order of incoming sounds in a sequence, a process that may be conserved in primates.

## Graphical abstract

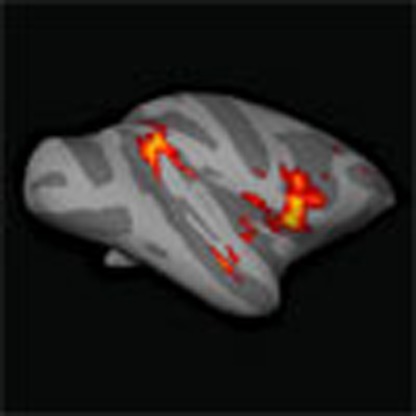

Central to debates about the neurobiological origins of language is the question of how far the neural systems supporting human language functions depend upon evolutionarily conserved processes that are also present in our primate relatives^1^,^2^,^3^. Human neuroimaging experiments have led to the development of a number of neurobiological models of language processing^4^,^5^,^6^ and hypotheses about the evolution of the brain areas that support these functions^2^,^4^,^7^,^8^,^9^. Testing such evolutionary hypotheses requires evidence from cross-species functional imaging studies, using paradigms that can both evaluate abilities present in human and non-human animals and which are known to engage language-related processes in the human brain.

Artificial grammars generate rule-based sequences of stimuli, which can be designed to investigate different types of sequencing computations^10^,^11^,^12^. Representative sequences are presented to the participant so that the statistical properties of the ordering relationships between the sequence elements can be learned, often referred to as statistical learning or sequence processing^13^,^14^. It is broadly accepted that these processes are linked to and important for understanding language learning^13^,^15^,^16^, as well as being useful for understanding the neurobiology of language and the frontal cortex^2^,^17^,^18^. This has led to a view of language acquisition involving a critical parallel, possibly implicit, process of statistical extraction^14^,^15^. Moreover, statistical learning has been shown to correlate with performance on language tasks and is impaired in agrammatic aphasic patients, suggesting that such sequence processing shares neural mechanisms with language-related processes in the human brain^16^,^19^. There are also a large number of studies showing that sound sequence analysis is a critical determinant of language disorder—so that ‘low-level’ auditory analysis is relevant to ‘high-level’ disease^20^,^21^. In addition, shared sequence processing capabilities have been identified in humans and non-human animals^8^,^11^,^22^,^23^,^24^, which are noted as candidate language precursor abilities in non-human primates^25^,^26^. Therefore, although language-specific processes are unique and can only be studied in humans, comparative sequence processing and statistical learning studies remain important for identifying language-related processes that are evolutionarily conserved (Supplementary Fig. 1 and Supplementary Note 1).

Human functional magnetic resonance imaging (fMRI) studies have provided further evidence for the link between sequence learning paradigms and language processing, demonstrating that certain sequencing operations can engage comparable brain areas to those seen in natural language tasks, including ‘perisylvian’ frontal, temporal and parietal areas in and around the Sylvian fissure or lateral sulcus^2^,^3^,^4^,^27^,^28^,^29^,^30^,^31^,^32^,^33^,^34^,^35^. Sequences containing violations of learned local ordering relationships, for example the transitions between adjacent elements in a sequence, engage ventral and opercular regions of frontal cortex^2^,^28^, which form a part of the ventral temporal to frontal lobe processing pathway^4^,^36^,^37^,^38^. These regions are also involved in processing similar short-distance grammatical relationships within or between adjacent phrases in natural languages^4^,^32^,^33^,^34^. Therefore, the ventral frontal and opercular cortex (vFOC) has been proposed to play a critical role in initial stages of human syntactic processing^4^. By comparison, sequences with more complex relationships between elements engage not only the vFOC but also the adjacent Brodmann Areas 44 and 45, which belong to a dorsal processing pathway^4^,^36^,^37^,^38^,^39^ and are involved in hierarchical processes in natural language^2^,^6^,^27^,^28^,^29^,^30^,^31^, such as the nesting of a phrase within another phrase in a sentence.

The combination of human neuroimaging and animal behavioural work on sequence learning has led to the hypothesis that the processing of sequences requiring the tracking of local relationships would engage vFOC comparably in humans and monkeys^2^,^4^,^7^,^8^,^9^. We conducted comparative fMRI sequence processing experiments in rhesus macaques and humans to test this hypothesis by assessing similarities and differences in how key ventral frontal brain regions, particularly vFOC and areas 44/45, might be recruited across species. We identified striking cross-species functional correspondences in the sequence processing functions of the vFOC. These results raise the possibility that language-critical processes in modern humans are functionally integrated with an ancestral, domain-general system that is involved in sequence processing.

## Results

### Macaque and human behaviour

**Figure 1:**
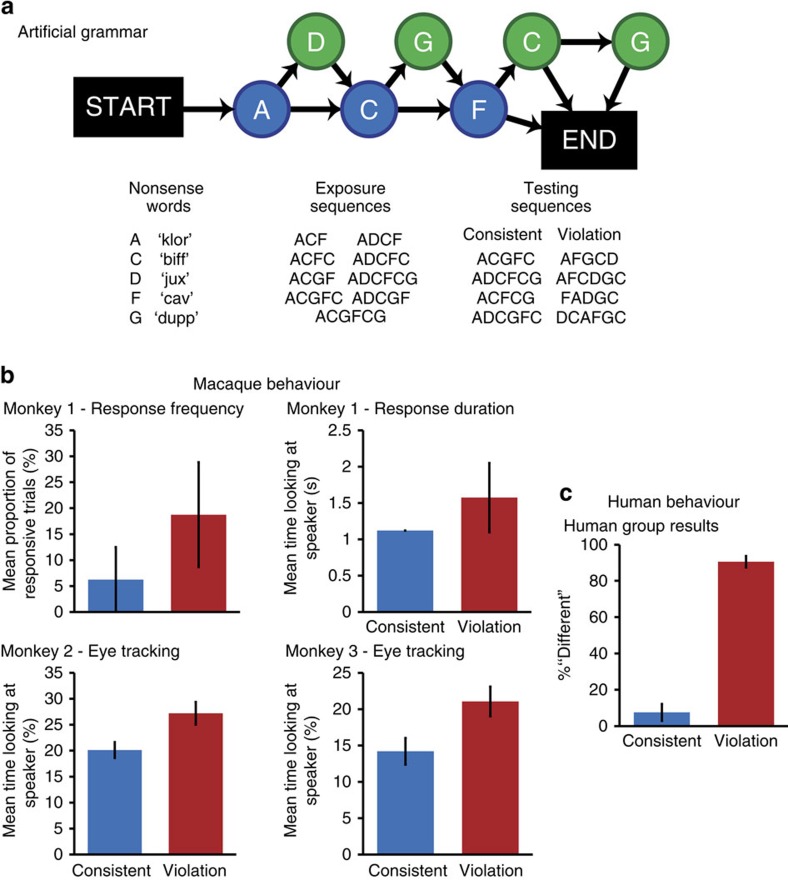
The artificial grammar and monkey and human behaviour. (**a**) The artificial grammar is represented by a feed-forward transition graph with non-deterministic branching points. Sequences are constructed from five auditory elements (nonsense words). Consistent sequences (strings of nonsense words) are generated by following any path of arrows from START to END. Violation sequences do not follow the arrows, for details see ref. 8. Experiments began with the participants being exposed to representative exposure sequences, followed by a testing phase evaluating responses to consistent and violation sequences. (**b**) Mean proportion of trials (±s.e.m.) on which the three Rhesus macaques made unambiguous looking responses towards a concealed audio speaker, which presented either consistent or violation testing sequences. Monkey 1 was tested with a video coding approach, as part of an experiment conducted with a group of 13 animals, and Monkeys 2 and 3 were also tested individually with eye-tracking, for further details see refs 8, 24. The macaques responded more frequently and for longer durations to violation sequences than the consistent ones. Monkey 1’s responses were representative of the significant effects seen in the group analyses (*t*_12_=7.898, *P*<0.001). Monkeys 2 and 3 each showed significantly more responses to the violation sequences (*t*_24_=3.137, *P*=0.004; *t*_24_=3.129, *P*=0.005). (**c**) Mean proportion of trials (±s.e.m.) in which 12 humans reported the testing sequences as ‘different’ relative to those heard during exposure (*t*_11_=15.437, *P*<0.001). For additional behavioural results, see Supplementary Figs 2 and 3.

In artificial grammar learning or sequence learning paradigms, participants are exposed to representative rule-based sequences that are ‘consistent’ with the artificial grammar, following which they are tested with sequences that are either ‘consistent’ or that contain illegal ‘violation’ transitions^10^. Stronger responses to sequences that violate the legal ordering of the elements relative to consistent sequences can provide evidence that implicit learning of the relationships between the stimuli in the sequences has occurred^4^,^12^,^14^. The current experiments used stimuli based on an artificial grammar paradigm previously used to test human infants^11^ and non-human primates^8^,^11^,^24^. The paradigm consists of branching relationships between several obligatory and optional elements (Fig. 1a). This produces considerable variability in the transitional probabilities between elements and emulates some of the properties of language and bird song^11^ (Supplementary Note 1). The stimuli consist of five auditory nonsense words that are arranged into sequences that are either ‘consistent’ with the ordering relationships or which contain illegal, ‘violation’ transitions between elements (Fig. 1a).

In a previous behavioural study, rhesus macaques (three of which participated in the current fMRI experiment) were exposed to representative, consistent sequences (Fig. 1a). In the following testing phase the animals showed stronger orienting responses to sequences containing violations of the ordering relationships, relative to novel, consistent sequences (Fig. 1b, (ref. 8)). Here we conducted a comparable behavioural experiment in humans and found that, after exposure, human participants showed similar behavioural sensitivity to the violation sequences (Fig. 1c). Additional analyses of the human and macaque behavioural data show that these results cannot be explained by simple learning strategies in either species, such as responding to highly salient violations of the ordering relationships early in the sequences (Supplementary Fig. 2). Instead, both species seemed to be sensitive to illegal transitions between elements throughout the sequences, and the effects generalized to novel consistent sequences not heard prior to the testing phase (compare effects for familiar versus novel consistent sequences in Supplementary Fig. 2). Furthermore, in both species we observed stronger responses to sequences containing more unexpected, unpredictable transitions (Supplementary Fig. 3). Altogether, the behavioural results demonstrate that the pattern of responses and strategies employed appear to be comparable across species (Fig. 1, Supplementary Figs 2 and 3), suggesting that macaques and humans respond similarly, based on the statistical probability with which the transitions between elements occur during the exposure phase.

### Macaque fMRI

**Figure 2:**
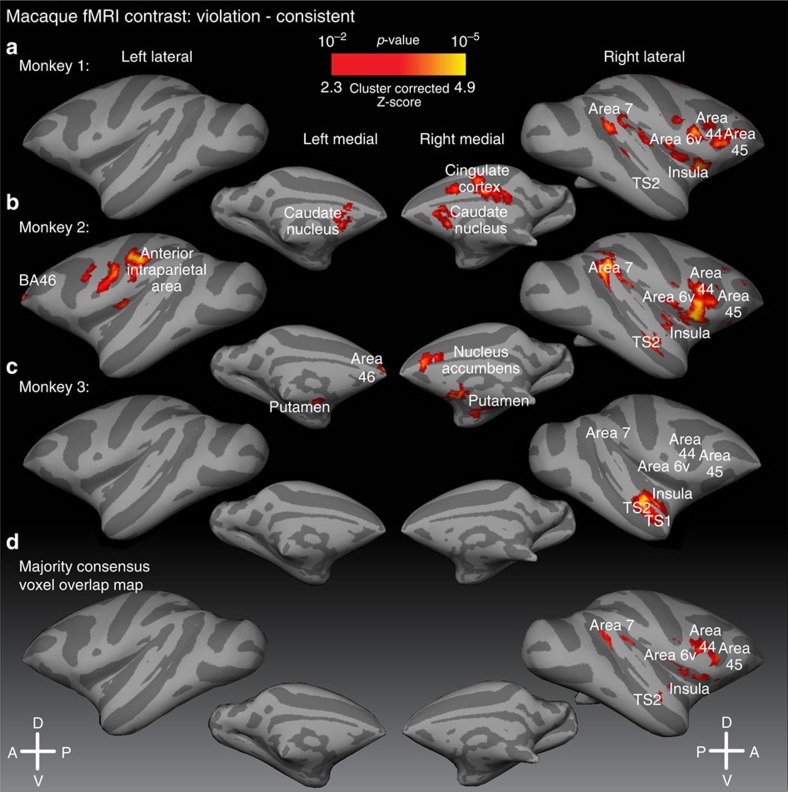
Macaque brain regions sensitive to sequence ordering violations. Statistical parametric maps of sensitivity to sequence violations (contrast: violation versus consistent) displayed in each of the three macaques (**a**–**c**) and in a majority consensus voxel-overlap map (**d**), all *P*<0.05 cluster corrected (see Methods). Results are displayed on rendered lateral and medial surface representations transformed to a standard monkey brain in register with a macaque stereotactic atlas (Methods); light grey: gyri; dark grey: sulci. A, anterior; D, dorsal; P, posterior; V, ventral, see also Supplementary Figs 5 and 6.

Functional MRI was used to reveal the brain regions associated with detecting sequence ordering violations in macaques and humans. Three rhesus macaques were first exposed to a representative set of consistent sequences (Fig. 1a), allowing them to implicitly learn the statistical properties of those sequences^14^,^16^ (Supplementary Figs 2 and 3). After the exposure phase, the macaques were then scanned with fMRI as they listened to testing sequences that were consistent with or that violated the rule-based ordering relationships (Fig. 1a; Methods). Figure 2 shows the brain regions sensitive to the violation sequences for each animal (contrast: violation versus consistent, see Methods and Supplementary Note 2) mapped onto a surface-rendered standard macaque template brain. Several significant clusters (*P*<0.05, cluster corrected) occurred in corresponding anatomical regions across the animals. Regions sensitive to sequence violations in all three animals included right ventral frontal cortex, involving opercular and dysgranular insular cortex, ventral to Areas 44/45 (Fig. 2). Even in monkey 3 (M3), who shows the more restricted pattern of activation of the three monkeys (Fig. 2c), there is clear engagement of the dysgranular insula in the frontal operculum (Supplementary Fig. 4). In a majority of the animals significant clusters of activation were also observed in additional ventral frontal regions (surrounding and including ventral area 6v), the anterior temporal lobe (for example, area TS2), and posterior parietal cortex area 7 (see Table 1 for a list of all the significantly activated brain areas).

**Figure 3:**
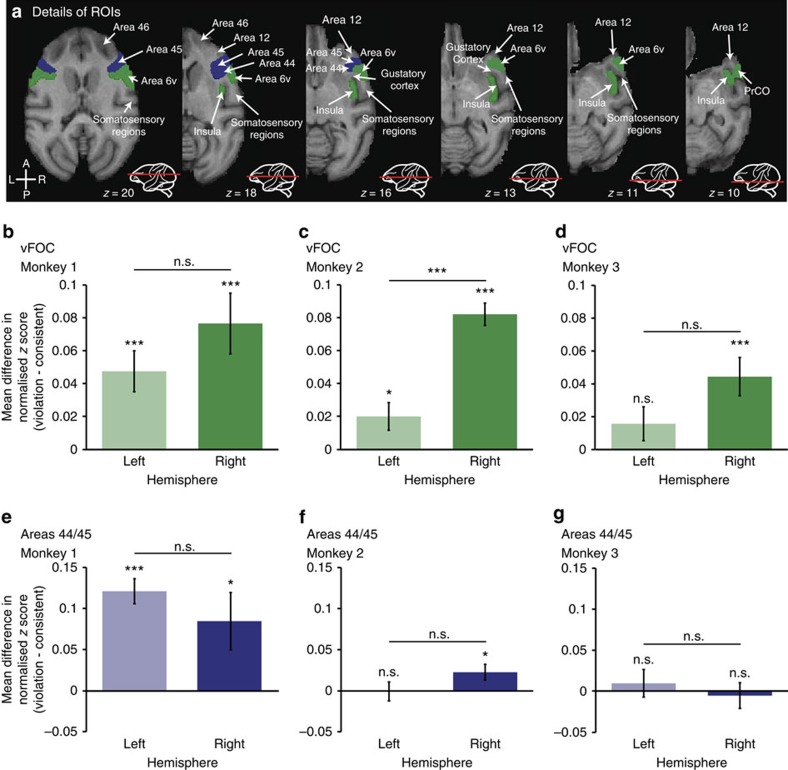
Macaque ROI results. (**a**) Bilateral anatomically defined ROIs used for analyses: blue comprises anatomical areas 44 and 45; green comprises adjacent vFOC areas, including anatomical areas PrCO, dysgranular insula and area 6v (Methods). Somatosensory and gustatory regions, and area 12 (orbital frontal cortex) were excluded. (**b**–**g**) Normalized mean fMRI response differences (violation versus consistent) in the vFOC and Areas 44/45 regions-of-interest shown by hemisphere in each of the macaques. Details of statistical analyses: One-sample *t*-tests with Bonferroni correction, in Monkey 1, left vFOC: *t*_366_=3.827, *P*<0.001; right vFOC: *t*_404_=4.155, *P*<0.001; Monkey 2, left vFOC: *t*_658_=2.401, *P*=0.034; right vFOC: *t*_702_=12.052, *P*<0.001; Monkey 3, left vFOC: *t*_632_=1.523, *P*=0.256; right vFOC: *t*_658_=3.818, *P*<0.001. Two-sample *t*-tests, between hemispheres in vFOC, in Monkey 1: *t*_770_=1.279, *P*=0.201; Monkey 2: *t*_1360_=5.771, *P*<0.001; Monkey 3: *t*_1290_=1.839, *P*=0.066. One-sample *t*-tests with Bonferroni correction, in Monkey 1, left areas 44/45: *t*_192_=8.000, *P*<0.001; right areas 44/45: *t*_181_=2.438, *P*=0.032; Monkey 2, left areas 44/45: *t*_276_=0.05, *P*=1.0; right areas 44/45: *t*_282_=2.372, *P*=0.036; Monkey 3, left areas 44/45: *t*_265_=0.583, *P*=0.561; right areas 44/45: *t*_270_=0.331, *P*=0.741. Two-sample *t*-tests, between hemispheres in areas 44/45, in Monkey 1: *t*_373_=0.977, *P*=0.329; Monkey 2: *t*_558_=1.556, *P*<0.120; Monkey 3: *t*_535_=0.653, *P*=0.514, see also Supplementary Figs 5 and 6. Symbols: n.s.,not significant; *P<0.05; **P<0.01; ***P<0.001.

These effects were further evaluated using planned region of interest (ROI) analyses. First, separate ROIs for the vFOC (vFOC, green ROI in Fig. 3a) and the adjacent areas 44/45 (blue ROI in Fig. 3a) were defined using accepted stereotactic coordinates for these regions in a macaque anatomical atlase (Methods). The vFOC ROI included ventral frontal cortical areas adjacent and inferior to areas 44/45, including the frontal operculum, ventral BA6v and dysgranular insular cortex, but excluding areas 44/45, much of area 47/12c (ref. 40) and all of area 49. A voxel-based repeated measures (RM) analysis of variance (ANOVA) was used to evaluate effects in the ROIs with the factors: Condition (consistent and violation sequences); ROI (vFOC, areas 44/45); Hemisphere (left, right); and Monkey (three levels). A significant main effect of condition (*F*_1,4886_=108.1, *P*<0.001) showed increased activation to violation relative to consistent sequences in these ROIs. There was no significant interaction between condition and ROI (*F*_1,4886_=1.149, *P*=0.284), suggesting that although macaque vFOC is strongly sensitive to sequence violations, and consistently so across the three animals, areas 44/45 are also involved to some extent. Regarding the lateralization of results, there was no significant interaction between Condition and Hemisphere (*F*_1,4886_=3.37, *P*=0.07), suggesting that the effects in these areas are not significantly lateralized to either hemisphere (see Supplementary Note 3 for a summary of lateralisation results). These analyses were complemented with analyses of the results in each of the animals individually (Fig. 3b–g). The individual animal results recapitulated the overall findings, showing significant activation to violation sequences relative to consistent sequences in the vFOC in all of the animals, and that statistically significant activation was also observed in areas 44/45 in two of the three macaques.

Two additional control experiments with two of the macaques further demonstrate that the vFOC response, (1) depends on prior exposure to representative, consistent sequences and (2) scales with the number of violations that occur in the testing sequences. Specifically, in the first control experiment, M1 was exposed to randomly generated sequences of nonsense words in which every transition between nonsense word elements occurred equally frequently. Following this randomized exposure, the animal was tested with testing sequences and scanning methods identical to those used in the original experiment (Figs 2 and 3). We confirmed that the auditory fMRI responses were matched in power between the original experiment and this control experiment, with both datasets showing a comparable sound versus silence response in auditory cortex (Supplementary Fig. 5a,b). In this control experiment, the previously noted vFOC response to the violation versus consistent testing sequences (Figs 2a and 3b,e) disappeared (Supplementary Fig. 5c,e). This observation demonstrates that the ventral frontal cortex response in the main experiments depends on exposing the animals to sequences containing predictable statistical relationships that can subsequently be used to identify the violation sequences. Therefore, the responses do not result from general deviance detection responses elicited by perceptual properties of the testing sequences (Supplementary Note 4). Rather, the violation sequences, by design, violate the ordering relationships between the sequence elements, which are established by the statistical regularities in the sequences heard during the exposure phase.

In the second control experiment, we scanned M2 with violation testing sequences each containing only a single violation (Supplementary Fig. 6) and compared these results to those with sequences containing multiple violations (Figs 1a, 2b and 3c,f). In comparison to the main experiment (Figs 2 and 3), here we observed weaker but similar activation of the vFOC, suggesting that the response of this region scales with the number of violations in the testing sequences and is evident at least to some extent even with single violations in sequences that are otherwise ‘consistent’ (Supplementary Fig. 6). Together these control experiments demonstrate a graded increase in the fMRI response in the vFOC. These results cannot be explained by a binary categorical or ‘deviant’ response, elicited by any sequence that contains a violation, and they provide further evidence that the fMRI response reflects the processing of the statistical properties of the sequences, learned during the prior exposure phase.

### Human fMRI

**Figure 4:**
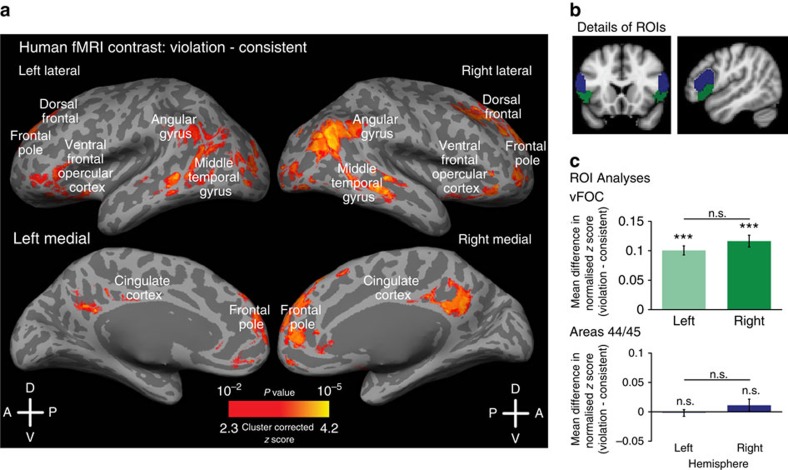
Human brain regions sensitive to sequence ordering violations. (**a**) Human group (*n*=12) statistical parametric map of sensitivity to sequence violations (contrast: violation versus consistent), all *P*<0.05 cluster corrected. Results are displayed on rendered medial and lateral surface representations (light grey: gyri; dark grey: sulci) transformed to the Montreal Neurological Institute standard brain and using human anatomical atlases (Methods). (**b**) vFOC (green) and areas 44/45 (blue) ROIs. (**c**) Normalized mean ROI voxel response differences (violation versus consistent) in the fMRI signal for vFOC and areas 44/45 in both left and right hemispheres of the human brain. Additional analyses to investigate activation in these two ROIs independently showed that the vFOC region was strongly sensitive to sequence violations bilaterally, while the areas 44/45 region showed no significant sensitivity to the violation sequences. One-sample *t*-tests with Bonferroni correction, in left vFOC: *t*_916_=12.930, *P*<0.001; right vFOC: *t*_971_=11.705, *P*<0.001; two-sample *t*-test between hemispheres in vFOC: *t*_1887_=1.244, *P*=0.214. One-sample *t*-tests with Bonferroni correction, in left areas 44/45: *t*_613_=−0.359, *P*=1.0; right areas 44/45: *t*_650_=1.064, *P*=0.576; two-sample *t*-test between hemispheres in areas 44/45: *t*_1262_=1.087, *P*=0.277, see also Supplementary Fig. 8. Symbols: n.s.,not significant; ***P<0.001.

Twelve human participants were exposed to the representative consistent sequences and then were scanned using fMRI while listening to the same consistent or violation stimulus sequences used to test the macaques (Fig. 1a; Methods). Large significant clusters (*P*<0.05, cluster corrected) that were sensitive to violation relative to consistent sequences (contrast: violation versus consistent; Fig. 4 and Table 1) were observed in both hemispheres in the ventral frontal cortex, including the frontal operculum, but was not evident in areas 44/45. Our results are consistent with previous findings showing that human vFOC is engaged in processing sequences with local adjacent transitions between elements^2^. Strong bilateral activations were also observed in the posterior parietal cortex BA39 and middle temporal gyrus, regions which have also been implicated in language processing^35^,^41^ (see Table 1 for the full list of significantly activated brain areas).

**Figure 5:**
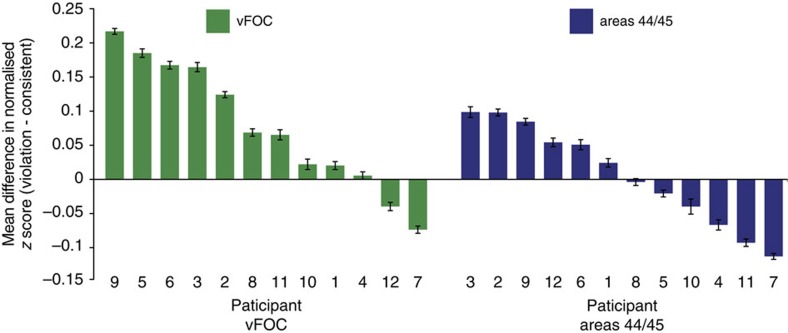
Individual human ROI results. Results of individual human ROIs from areas (**a**) vFOC (green) and (**b**) areas 44/45 (blue), shown as the difference in activation to the ‘violation’ minus ‘consistent’ sequences in each of the 12 human participants individually, ranked by mean activation across both ROIs (±s.e.m.). The vFOC shows a more consistent sensitivity to violations of the sequencing relationships than the areas 44/45 ROI, see text. Possibly due to the high levels of task performance by all of the human participants (mean performance=91%, s.d.=2.7%), no significant correlation was observed between behavioural performance and activation in either the vFOC (*r*=−0.19, *P*=0.56) or areas 44/45 (*r*=0.11, *P*=0.73) ROIs.

To evaluate the effects in vFOC in relation to the adjacent areas 44/45 and to allow direct cross-species comparisons, we conducted planned ROI analyses identical to those performed in the macaques. An ROI including areas 44 and 45 (blue in Fig. 4b) was defined using accepted atlases of human anatomical regions in stereotactic coordinates (see Methods). A vFOC ROI (green in Fig. 4b), adjacent and inferior to areas 44/45, was also defined, including the frontal operculum, insular cortex and additional ventral frontal regions, but excluding areas 44/45 and much of BA47. As in the macaques, a voxel-based RM-ANOVA was used to evaluate effects in the ROIs, with the factors: condition (consistent and violation sequences), ROI (vFOC, areas 44/45) and hemisphere (left, right). A significant main effect of condition showed strong fMRI activity for violation sequences in these regions (*F*_1,3149_=150.478, *P*<0.001). The sensitivity to violation sequences was not significantly lateralized, as shown by no interaction between condition and hemisphere (*F*_1,3149_=2.469, *P*=0.116). Greater activation to violation sequences occurred in the vFOC relative to the areas 44/45 region, shown by an interaction between condition and ROI (*F*_1,3149_=127.353, *P*<0.001). These results were supported by separate analyses of the effects in these two ROIs (Fig. 4c). As in the macaques, these analyses confirm the central role of vFOC in both hemispheres in detecting AG sequence violations. These data also suggest that human areas 44/45 are not significantly engaged by violation sequences at the group level. Conducting individual human ROI analyses, as was done with the macaques, shows that the vFOC sensitivity to violations of the sequence ordering relationships is robust in most (9/12) of the human participants but that the effect in areas 44/45 is more variable across participants, being evident in six of the twelve human participants (Fig. 5).

### Macaque and human fMRI comparisons

The human and monkey ROI results were directly compared using an RM-ANOVA with the factors: condition (consistent, violation), species (human, monkey), ROI (areas 44/45, vFOC) and hemisphere (left, right). This showed a significant overall sensitivity in the ROIs to violation relative to consistent sequences (main effect of condition: *F*_1,8043_=234.6, *P*<0.001). A significant condition by ROI interaction was observed (*F*_1,8043_=90.452, *P*<0.001) confirming that the vFOC is more strongly activated than areas 44/45 across both species. A two-way interaction was observed between condition and species (*F*_1,8043_=8.277, *P*=0.004), indicating that the human brain is relatively more sensitive to the sequence ordering violations than the macaque brain. Furthermore, a significant interaction between condition, ROI and species was observed (*F*_1,8043_=52.2, *P*<0.001), showing that areas 44/45 were statistically more involved in the monkeys than in the humans. These cross-species RM-ANOVA results for the vFOC and areas 44/45 ROIs are supported by two separate RM-ANOVAs including each ROI separately (Supplementary Note 5). Note, however, that the individual monkey (Fig. 3) and human (Fig. 5) ROI analyses show areas 44/45 to be more variable in effect across participants. The vFOC effects we observe are robust and stable in response to the violation sequences across humans and monkeys. These observations suggest substantial correspondence between the macaque and the human results associated with sequence processing, especially in vFOC.

## Discussion

This study provides evidence that regions of vFOC are comparably functionally engaged in monkeys and humans by sequences that violate the ordering relationships of an auditory artificial grammar^8^,^9^. We used a comparative sequence processing paradigm and fMRI to provide a first test of a prominent neurobiological hypothesis about the evolution of human brain regions involved in initial syntax-related processes between words in a sentence or evaluating adjacent sequence elements^2^,^4^,^7^,^9^. Since non-human animals lack human language abilities, the results of this comparative human and monkey fMRI experiment inform us on the domain-general processes, not specific to language, which macaques and humans both possess and whose neural substrates are identified. The results of these comparative analyses provide evidence that regions of vFOC support evolutionarily conserved sequence processing functions. Our human fMRI results also link to the larger body of evidence that the frontal opercular cortex is associated with certain forms of human language processing and domain-general processes, as we consider.

vFOC is argued to represent an initial stage in neurobiological processes related to human syntax^4^. This region is involved in processing relationships within and between adjacent phrases in natural language^4^,^32^,^33^,^34^. Sequences that violate an artificial grammar designed to model similar adjacent syntactic relationships in natural language^22^ also engage these ventral frontal and opercular human brain areas^2^,^28^. Thus, prior human neuroimaging studies have suggested that the vFOC , which lies adjacent to but does not include areas 44/45, is involved in ‘local structure building’, thought to represent a key initial stage of human language processing^2^,^4^,^7^. By contrast, sequences that violate more complex sequence relationships engage this region and also areas 44/45 (refs 2, 27, 28, 29, 30, 31). Areas 44/45 are particularly engaged by syntactically and semantically complex natural language processes^4^,^5^,^6^,^42^,^43^ as well as other complex domain-general sequencing processes^3^,^44^. In parallel, non-human primates have been shown to be able to detect illegal, violation transitions between at least adjacent sequence elements^8^,^11^,^22^,^24^. There is also some evidence that New World monkeys and apes are sensitive to non-adjacent relationships^45^,^46^,^47^, although there is a paucity of evidence that any non-human animals are able to recognize sequences which model more of the complexity of human language. These studies led to the novel hypothesis, tested here, that local sequence processing abilities, and the ventral frontal opercular regions that support them in humans, might be functionally conserved in certain extant non-human primates^2^,^4^,^7^, reflecting a common evolutionary origin.

Our fMRI results reveal that ventral and opercular regions of frontal cortex are comparably involved in auditory sequence processing operations in humans and macaques. This vFOC activation was statistically robust, and was consistently observed across all three macaques and in the human participants at the whole brain and ROI levels, including with individual monkey and human ROIs (Figs 2, 3, 4, 5, Supplementary Figs 4 and 5). These results suggest that the sequence processing function of the vFOC is shared by humans and macaques. The role of areas 44/45 in this task was variable both within and between species. Activation in this region was observed in two out of the three macaques (Fig. 3e,f). No significant activation was seen at the group level in humans (Fig. 4c), however, individual analyses revealed that areas 44/45 were engaged in 6 of the 12 human participants (Fig. 5). Furthermore, in the human RM-ANOVA group results, the vFOC ROI was significantly more responsive to the violation sequences than the areas 44/45 ROI, and the across species RM-ANOVA analyses showed an interaction indicative of significantly greater areas 44/45 ROI engagement in the macaques relative to the humans in response to violations of the sequencing relationships. It remains possible that if more human participants were tested significant activation might have been observed in human areas 44/45 at the group level. However, we are careful not to over interpret the variable activation pattern in areas 44/45 in the current experiments. Therefore, the primary observation in this study is the statistically robust activation in the vFOC in both the humans and monkeys. These results provide evidence supporting the hypothesis that vFOC is comparably involved in sequence processing in macaques and humans. This region appears to be specifically involved in evaluating the transitions between sequence elements, in the context of the regularities learned during the prior exposure.

To test the frontal operculum evolutionary hypothesis^4^ it was necessary to select a sequence processing task with an appropriate level of complexity, which both humans and macaques learn^48^. In this study, we used a moderately complex sequencing paradigm that can generate a range of legal sequences of different lengths, containing a wide range of transitions between elements (Fig. 1a, ref. 8). Our human fMRI results show that these sequencing operations are complex enough to activate ventral frontal opercular regions, consistent with earlier human fMRI studies of adjacent sequencing^2^,^28^. Furthermore, our behavioural results demonstrate a striking correspondence between the responses of the macaques and the humans (Fig. 1b,c, Supplementary Figs 2 and 3), suggesting that (unlike the behavioural pattern we previously observed in another species of monkey, common marmosets^8^) both macaques and humans produce a similar pattern of responses. Neither the responses of the humans or the macaques can plausibly be attributed to reliance on simple cues (such as the novelty of the testing sequences, Supplementary Fig. 2), or by trivial acoustical differences between the consistent and violation sequences (Supplementary Fig. 5). Furthermore, we demonstrated that both the humans and macaques respond more strongly to sequences containing less predictable transitions, suggesting that both species track the statistical properties of the transitions throughout the sequences (Supplementary Fig. 3).

The form of sequence processing required by this paradigm is markedly different to ‘deviance detection’ or ‘oddball’ responses elicited by the appearance of an unexpected sound or pattern of sounds in a stream of auditory stimuli^49^. In these tasks detecting the deviant stimuli involves noticing a perceptual difference in the stream. By comparison, our paradigm contains no ‘oddball’ sounds in any of our sequences: the same five nonsense word elements appear in both the violation and consistent sequences. Therefore none of the individual elements represent ‘deviants’ in the same way as in oddball tasks; only the ordering relationships between the same set of elements is regulated or violated. Furthermore, the two types of paradigms appear to engage different brain networks^49^ (Supplementary Note 6).

A number of studies highlight the correspondences that can be drawn between the prefrontal cortex in humans and non-human primates, as well as current areas of uncertainty. The earlier comparative neuroanatomical studies by Brodmann and more recent efforts have identified anatomical correspondences between many prefrontal regions, such as between areas 44 and 45 in humans and non-human primates^50^,^51^. Other active fields of research involve understanding the comparative connectivity of frontal and other areas that in humans are known to be involved in language-related processes^39^,^52^. Some authors have predicted that during human language evolution the interconnectivity between frontal and other areas increased^53^, which also predicts functional differences in otherwise cytoarchitectonically corresponding areas, such as area 44 (ref. 54). Our comparative behavioural and fMRI results in humans and macaques contribute to these efforts by providing functional insights on ventral frontal cortex regions, highlighting the role of the frontal operculum, which is a less well studied region of the frontal cortex. We also present results using both anatomically defined ROI analyses (Figs 3 and 4) and whole-brain analyses, unconstrained by any particular parcellation scheme (Figs 2 and 4, Table 1), highlighting the correspondences in activation patterns at particular locations in the brains of the monkeys and humans across the species. This information could now be used as seed regions to determine the cross-species correspondences in anatomical pathways that are currently under question^37^,^55^,^56^.

Although many language-related processes are left-lateralized in humans (especially those involving complex syntax), these experiments revealed no significantly left-lateralized effects in either species. While some of the results are suggestive of potential right lateralization, statistical testing failed to provide consistent evidence that sequence processing in the key frontal areas is significantly lateralized in either macaques or humans. For a summary of the observations on lateralisation effects see Supplementary Note 3. Previous hypotheses about the evolutionary origins of the functions supported by the ventral frontal cortex have primarily focussed on the left hemisphere^2^,^4^,^7^. However, the human and macaque results reported here suggest that the right hemisphere also plays an important role in sequence processing. More generally, these observations of a lack of strong lateralization provide the first direct comparative evidence in support of a ‘dual neurobiological language systems’ hypothesis^6^,^57^,^58^, but extend it in important ways. This hypothesis proposes that in modern humans, specializations for core syntactic language functions depend on a left-lateralized ventral fronto-temporal system, and that this left-lateralized system is functionally integrated with a more bilaterally distributed network, that has more general language-related functions, such as those that combine semantic and syntactic operations in natural language^6^. Given that our results are based on an artificial grammar or sequence learning paradigm^8^,^9^, the current findings suggest that the hypothesized bi-hemispheric system also supports sequence processing in both humans and monkeys, forming an important part of the ancestral systems that underpin aspects of language function in the modern human.

The neurobiological hypotheses that we tested here focussed on vFOC and areas 44/45. However, we also observed activation in broadly comparable perisylvian regions, including parietal regions, in the humans (Fig. 4) and two out of three of the monkeys (Fig. 3). See Supplementary Fig. 7 for ROI results from this region in humans and monkeys. The focus of the parietal clusters that we observed appears to involve BA7 in macaques and BA39 in humans, both of which lie below the intraparietal sulcus. There has been considerable uncertainty as to which dorsal parietal regions are functionally homologous^59^, given that human BA7 lies above the intraparietal sulcus. Our results suggest a level of functional correspondence between BA7 in macaques and BA39. Relatedly, MRI-based estimates of connections in the inferior parietal lobule in humans and macaques have shown similarities between different aspects of the inferior parietal lobule in the two species^60^. It is interesting to note that in the human brain this parietal region is engaged by a wide range of language tasks^4^,^32^,^61^,^62^. However, neuropsychological and neuroimaging evidence (for example, ref. 41) suggests that the human inferior parietal lobule, although providing a substantial role in sentence comprehension, is not as critical to the core syntactic language functions as the perisylvian inferior/ventral frontal regions. Therefore, the precise role of the parietal region in our task might well differ in relation to the role of the vFOC in both species, and would need to be further studied in the future.

It is also important to consider the impact of differences in the way the species were tested on the results, since it is not possible to conduct all measurement in the same way with both species. The behavioural results show that despite different testing methods after the exposure period, both species responded similarly to the statistical properties of the test sequences (Fig. 1, Supplementary Figs 2 and 3). Furthermore, fMRI data obtained from human participants scanned using the same fixation task as the macaques produced activation in the ventral frontal cortex that is very similar to the effects observed using the button press task in the main human experiment (compare Fig. 4 with Supplementary Fig. 8), and also to the macaque experiment (Figs 2 and 3). While it is possible that these or other testing differences (for example, the macaques and humans, respectively, received either juice or money as reward for participating) could obscure potential similarities between the species, the highly consistent results we observe in vFOC, in both species and in all of the monkeys, are remarkable given the inevitable methodological differences in how the species were tested (Supplementary Note 7). Our data suggest that the engagement of vFOC in both species is a key property of a common neural response to the violation of the sequencing relationships, and that this is present independent of the responses required by different tasks.

The extent to which language-specific and cognitive-domain-general processes overlap and share common processes in the human brain is an active area of research that informs us on the function of the frontal cortex. For example, Fedorenko *et al.*^3^, have suggested that domain-general subregions of area 44 lie next to regions that are more involved in language-specific functions^3^. Other studies have shown that the human inferior frontal gyrus and operculum are involved in cognitive processes such as attention and task control^44^,^63^,^64^. The type of comparative study that we conducted can help to link these disparate strands of research (Supplementary Fig. 1) and provide important inputs into future developments. For example, human neuroimaging studies aiming to distinguish domain-general from language-specific processes often use hierarchically organized natural language material. Our study points to the greater need for such studies to compare sequence processing tasks of different levels of complexity, some of which, as we show, can now be linked to a primate model system. Sequence processing paradigms can also readily incorporate visual or auditory stimuli and thus inform us on sequencing processes that are sensory modality independent. Moreover, although we found little evidence for non-specific deviance-detection responses in any of our results (Supplementary Note 6), such processes may well engage the human and monkey vFOC once transitional regularities are encoded. Future studies might tease apart the contributions of different cognitive processes involved in various forms of sequence processing. Statistical or sequence learning paradigms remain appealing because they can create specific prediction errors (violations) in sequencing relationships (Supplementary Fig. 6; also see ref. 65) that can be studied at the neuronal level in animal models and, as we show, linked to processes in humans by way of comparative behaviour and neuroimaging (fMRI or electroencephalography^65^).

In summary, the results of these experiments provide the first evidence supporting the hypothesis that functions of key regions of vFOC are evolutionarily conserved in macaques and humans. These regions play an important role in sequence processing, and, as shown in a number of prior human neuroimaging studies, are also involved in analysing local grammatical relationships in the human brain. We conclude that vFOC in both species supports neuro-computational functions which include evaluating sensory sequences in relation to the probabilities with which the transitions between elements previously occurred. This raises the possibility that language-related processes in modern humans are functionally integrated with highly conserved, originally non-linguistic processes shared with our extant primate relatives.

## Methods

All animal work and procedures performed were approved by the Animal Welfare and Ethical Review Body at Newcastle University and by the U.K. Home Office. The work complies with the Animal Scientific Procedures Act (1986) on the care and use of animals in research, and with the European Directive on the protection of animals used in research (2010/63/EU). We support the principles on reporting animal research stated in the consortium on Animal Research Reporting of *in vivo* Experiments (ARRIVE). All persons involved in this project were Home Office certified and the work was strictly regulated by the U.K. Home Office. Human participants provided informed consent to participate in this study, which was approved by the human studies Ethical Review Body at Newcastle University and which conformed with the 2013 WMA Declaration of Helsinki.

### Stimuli

Each of the stimulus sequences (see Fig. 1a) was constructed by digitally combining recordings of naturally spoken nonsense words (that is, the elements in the artificial grammar sequences) produced by a female speaker and recorded with an Edirol R-09HR (Roland Corp.) sound recorder. The amplitude of the recorded sounds was r.m.s. balanced and the nonsense word stimuli were combined into sequences using customized Matlab scripts (100-ms inter-stimulus intervals). The experiments were coded in Matlab (Psychophysics Toolbox: http://psychtoolbox.org/) and Cortex software (Salk Institute). All sounds were presented at ∼75 dB SPL (calibrated with an XL2 sound level meter, NTI Audio). For details on the sound delivery for the behavioural experiments, see: ref. 8. During scanning of both humans and monkeys, sound delivery was achieved through MRI-compatible headphones (NordicNeuroLab). The consistent and violation sequence were balanced for duration and element composition, for details see ref. 8. Briefly, the duration of the naturally spoken nonsense word stimuli within the sequences was as follows: Klor=0.64 s; Jux=0.62 s; Cav=0.56 s; Biff=0.40 s; Dupp=0.39 s. The consistent and violation sequence sets were balanced in the number of elements in the sequences (Fig. 1a) and the mean lengths (±s.d.) of the sequences were comparable: consistent sequences, 3.14 (0.42) sec; violation sequences, 3.25 (0.28) sec. We confirmed that there was no significant difference in sequence sound duration between consistent and violation sequences (independent samples *t*-test, *t*_6_=0.44, *P*=0.68), or in the duration of the individual elements present in consistent versus violation sequences (*t*_42_=0.61, *P*=0.55; ref. 8).

### Macaque behavioural experiment 1: video coding

Thirteen rhesus macaques (age range: 4–16 year-old) in two group housed colonies were exposed to the nine exposure sequences presented in random order for 2 h the evening before the experiment (Fig. 1a). The following morning they were refamiliarized with these sequences for 10 min. They were then presented with 32 test sequences (4 randomized repetitions each of the 4 consistent and 4 violation sequences, Fig. 1a) from a concealed audio speaker. The macaques’ looking responses towards this speaker were video recorded. These responses were all independently blind-coded offline by three raters using a Likert scale. Orienting responses were calculated based on the proportion of trials on which at least two out of three of the raters agreed that an unambiguous response, caused by the stimulus presentation, had occurred^8^.

### Macaque behavioural experiment 2: eye-tracking

Three rhesus macaques (aged 6, 6 and 14-year-old) that had previously participated in the video-coding behavioural experiment were individually tested in an eye-tracking experiment using a head post for head immobilization and infra-red eye-tracking system (Arrington Research Inc.), for additional details see: ref. 8. Following 30 min of exposure to representative consistent sequences (Fig. 1), the animals participated in a simple fixation task. After fixating on a central fixation spot for 2 s to obtain a juice reward, the fixation spot would disappear and on 25% of trials the animals were presented with a randomly selected test sequence presented from either the right or left audio speaker. Looking responses toward the presenting speaker were recorded and the durations of these responses were analysed^8^. No feedback of any sort was given to the macaques for their behavioural responses to the testing sequences.

### Human behavioural experiment

Twelve human participants (mean age 23; 6 female, 6 male, all right handed) were exposed for 5 min to the same exposure sequences as the monkeys (stimuli and rate of presentation were identical between experiments; Fig. 1). The participants then listened to the testing sequences (Fig. 1a). Following each testing stimulus sequence presentation the human participants responded by using button presses to classify the test sequence heard as either following the ‘same’ pattern as the exposure sequences (consistent) or a ‘different’ pattern (violation). No feedback of any sort was given to the participants for their behavioural responses to the testing sequences.

### Macaque MRI procedures

Measurements of the fMRI blood oxygen level dependant (BOLD) signal were made on a non-human primate dedicated, vertical 4.7 T research MRI scanner (Bruker BioSpin, Etlingen, Germany). The monkeys sat in a primate chair in the scanner. To minimize the impact of head movements on the MRI results, the subjects were acclimated to periods of head immobilization using a head post that had previously been surgically implanted under anaesthesia and in aseptic conditions (for additional details see refs 66, 67). Signals were acquired using a birdcage RF coil. Functional data were acquired using a gradient-recalled echo planar imaging sequence with the following typical parameters: echo time, (TE): 21 ms; sparse imaging, volume acquisition time: 2 s, volume repetition time: 10 s; flip angle: 90°; 16 slices, 2 mm thickness; in-plane field of view: 12.8 × 9.6 cm, on a grid of 128 × 96 voxels, with a voxel resolution of 1 × 1 × 2 mm. Anatomical images were acquired in register with each functional scanning experiment using a 3D T1-weighted MDEFT sequence with parameters TE: 6 ms; repetition time: 750 ms; inversion delay: 700 ms; 22 slices; in-plane field of view: 12.8 × 9.6 cm^2^, on a grid of 256 × 192 voxels, with voxel resolution of 0.5 × 0.5 × 2 mm; number of segments: 8.

Three macaques were scanned for this experiment. All three of the macaques had participated in behavioural Experiment 1 (M1, M2 and M3, Fig. 1), and two in behavioural Experiment 2 using eye-tracking (M2 and M3). All the animals were male, aged 9, 14 and 6 years, respectively, weighing 6–12 kg, and lived in a colony of pair housed macaques. The animals were fMRI trained to complete trials of an established sparse-imaging/stimulation sequence with visual fixation and without requirement of an explicit response during the auditory stimulation^66^,^67^. Each scanning session began with a 30 min exposure phase where the exposure sequences were presented binaurally over headphones (NordicNeuroLab) in a randomized order (rate of 9 sequences/min; inter-sequence interval=4 s; Fig. 1). The fMRI scanning was conducted using a sparse-imaging fMRI paradigm^66^, whereby the stimuli could be presented in relative silence and the stimulus-related volume followed each stimulus sequence by 3.5 s to account for the hemodynamic response lag^68^. In the scanner, the animals were presented with a randomized sampling of consistent and violation test sequences (40% each type of trial, Fig. 1) or silent trials (20% of trials). Each scanning run consisted of 30 trials, before which the monkeys were re-familiarized with the exposure sequences for 3 min. The number of trials obtained in each animal depended on their motivation to participate in the fixation trials for a juice reward. Unexpected animal-related issues resulted in final datasets with different numbers of completed trials (M1: 160 trials; M2: 1410 trials; M3: 780 trials). Nonetheless, all three animals showed significant cluster corrected activity responses, including several clusters for M1 who had completed the fewest trials. We also noted that that there was no obvious relationship between the number of scanning runs and the sound versus silence response in auditory cortex (*r*=0.11, *P*>0.9), or the violation versus consistent contrast in the vFOC (*r*=−0.30; *P*>0.7).

### Macaque fMRI data analysis

For each animal we performed a first-level, fixed-effects General Linear Model analysis (FEAT, FSL) contrasting BOLD responses to violation versus consistent sequences. This involved motion correction and registration of the functional images to the macaque’s own high resolution anatomical scan, via intermediate scans including a lower resolution anatomical scan that was in register with the functional scans. The results of the first-level GLM analyses were then registered to a standard macaque template brain^69^, which is in register with an accepted macaque atlas in stereotactic coordinates, to determine the anatomical fields within which the significant activity clusters occurred^40^. The results were then combined into higher level analyses, evaluated at the cluster corrected (*P*<0.05) level (2 mm smoothing full-width half maximum, FWHM). The results were then registered to a FreeSurfer (http://surfer.nmr.mgh.harvard.edu/) surface-based representation of a standard template monkey brain^69^. For display purposes, a majority consensus map was used to summarize the macaque whole-brain results (Fig. 2d). This was created by identifying common voxels which were significantly activated (*P*<0.05 cluster corrected) in at least two of the three animals and plotting the average *z*-score values on the rendered surfaces.

ROIs were anatomically defined in reference to a *R. macaque* atlas of anatomical regions in stereotactic coordinates^40^. One ROI consisted of areas 44 and 45. The other ROI consisted of the vFOC, including areas PrCO, area 6v and the dysgranular insula in the frontal operculum (see Fig. 3a). Excluded from this ROI were somatosensory areas 1, 2 and 3, the gustatory cortex, areas 44/45 and orbital frontal areas. These ROIs were then registered back to the animals’ own functional imaging data. This helped to avoid overinflating the number of voxels used for analysis (than if the higher resolution standard anatomical brain had been used). However, in this case, given the functional imaging scan resolution of 1 × 1 × 2 mm^3^ where the slice thickness is greater than the in-plane resolution, the animals’ results presented with some differences in the numbers of voxels in each ROI available for analysis. The average BOLD signal in response to violation versus consistent sequences was calculated for each voxel in the ROIs. These values were normalized based on the maximum activation observed in either ROI in each animal. These data were analysed with RM-ANOVAs and *t*-tests using Bonferroni corrections for multiple comparisons.

### Human MRI procedures

Measurements of the fMRI BOLD signal were made on a human dedicated clinical/research 3 T scanner (Phillips). Functional data were acquired using a gradient-recalled echo planar imaging sequence with the following typical parameters: echo time, TE: 30 ms; sparse imaging, volume acquisition time: 2 s, volume repetition time: 10 s; flip angle: 90°; 28 slices, 4.5 mm thickness; in-plane field of view: 19.2 × 19.2 cm^2^, on a grid of 64 × 64 voxels, with voxel resolution of 3 × 3 × 4.5 mm. T1-weighted anatomical images were acquired in register with each functional scanning experiment with parameters TE: 4.6 ms; repetition time: 9.6 ms; 150 slices; in plane field of view 25.1 × 25.1 cm^2^, on a grid of 288 × 288 voxels; with voxel resolution of 0.87 × 0.87 × 1.2 mm.

The twelve participants (six female, six male) who took part in the behavioural experiment were scanned in the fMRI experiment between 1 and 2 weeks following behavioural testing. Before scanning the participants were presented with the exposure sequences in a randomized order for 10 min (rate of nine sequences permin; inter-sequence interval=4 s; Fig. 3a). In the scanner the participants were presented with the same consistent and violation test sequences as the macaques (40% each of type of trial, Fig. 3) and silent trials (20% of trials). As in the behavioural experiment, participants were asked to respond ‘same’ or ‘different’ using an MRI-compatible response box during the fMRI scanning. Each scanning run included 50 stimulus trials (200 trials total for each participant). Following each of the scanning runs (50 trials) the participants were re-exposed to the exposure sequences for 2–3 min before continuing with the next scanning run.

### Human fMRI data analyses

We performed first-level, fixed-effects General Linear Analysis (FEAT analysis implemented in FSL) contrasting BOLD responses to violation versus consistent sequences at the individual level and registered to a standard template human brain (Montreal Neurological Institute, standard in FSL). The individual data were then analysed using a higher level mixed-effects group analysis using a cluster corrected (*P*<0.05) threshold level (5 mm smoothing, FWHM). Results were then registered and projected to a Free Surfer (http://surfer.nmr.mgh.harvard.edu/) surface-based representation of a template human brain. The results were highly comparable when the data were analysed using a fixed-effects model. Table 1 also shows that the clusters (see peak *z* scores and *P* values) survive higher significance thresholds. No gender differences were observed in the fMRI results (Supplementary Note 8).

ROI analyses were conducted as follows. ROIs for Areas 44/45 and vFOC in both hemispheres were defined on the standard brain. The human anatomical regions were defined using human atlases of anatomical regions in stereotactic coordinates (Harvard-Oxford Cortical Structural Atlas and the Juelich Histological Atlas^72^) (ref. 70). The vFOC ROI was defined encompassing parts of the inferior frontal cortex ventral to areas 44/45, including the frontal operculum and the insula, but excluding areas 44 and 45 (Fig. 4). To ensure the results were comparable across species, the average BOLD activation in each ROI was normalized and analysed in the same way as the macaque data.

## Additional information

**How to cite this article:** Wilson, B. *et al.* Auditory sequence processing reveals evolutionarily conserved regions of frontal cortex in macaques and humans. *Nat. Commun.* 6:8901 doi: 10.1038/ncomms9901 (2015).

## Supplementary Material

Supplementary Information Supplementary Figures 1-8, Supplementary Notes 1-8 and Supplementary References

## Figures and Tables

**Table 1:** Anatomical locations of significantly activated clusters in *R. macaques* and human participants.

***R. Macaques***						
**Anatomical location**	**Stereotactic coordinates**			**Cluster corrected max.** ***z*** **score**	**Cluster*****P*** **value**	**Hemisphere**
	**x**	**y**	**z**			
*Monkey 1*						
Ventral frontal cortex	25	26	20.5	3.55	6.04 × 10^−6^	Right
Area 45, area 44, dysgranular insula, area 6, putamen						
Temporo-parietal regions	14.5	8	17.5	4.16	1.03 × 10^−3^	Right
Area 7, caudate nucleus (posterior), posterior auditory cortex (Pro, CM)						
Cingulate cortex	8	14.5	25	3.08	3.46 × 10^−3^	Right
Anterior/posterior cingulate cortex, caudate nucleus						
Caudate nucleus	1.5	24	18	3.08	3.41 × 10^−2^	Bilateral
						
*Monkey 2*						
Ventral frontal cortex and anterior temporal lobe	27	21.5	11.5	4.89	1.3 × 10^−3^	Right
Area 6v, dysgranular insula, anterior auditory cortex (TS2), somatosensory regions 1 and 2						
Frontal pole	−4	24.5	20	3.93	3.86 × 10^−4^	Left
Anterior intrapartietal area	−19.5	12.5	29.5	3.88	1.83 × 10^−2^	Left
Anterior and ventral intraparietal areas						
Caudate nucleus and putamen	5	32.5	20.5	3.87	1.45 × 10^−2^	Right
Temporo-parietal regions	16	1.5	25	3.52	1.21 × 10^−4^	Right
Area 7, posterior auditory cortex (TPT)						
Nucleus accumbens	2.5	20.5	7	3.49	4.23 × 10^−2^	Bilateral
Auditory cortex and insula	22	16	10.5	3.10	2.61 × 10^−4^	Right
Auditory cortex (R, RM), dysgranular insula, putamen						
						
*Monkey 3*						
Frontal opercular insular and temporal cortex	26	19	7.5	3.60	3.17 × 10^−2^	Right
Anterior auditory cortex (TS1, TS2), dysgranular insula, frontal operculum						
Majority consensus (overlapping significant voxels in 2+ monkeys)						
Dysgranular insula	20.5	17	17			Right
Area 6v	24.5	27	19.5			Right
Anterior auditory cortex (TS2)	26.5	20.5	11.5			Right
Area 7	12	0.5	23			Right
Caudate nucleus and putamen	14.5	24	17			Right

**Humans**						
**Anatomical location**	**MNI coordinates**			**Cluster corrected max.** ***z*** **score**	**Cluster*****P*** **value**	**Hemisphere**
	***x***	***y***	***z***			
Ventral frontal cortex	46	22	−12	3.55	1.90 × 10^−4^	Right
Frontal opercular cortex, frontal orbital cortex, frontal pole						
Ventral frontal cortex	−40	34	−10	3.26	5.61 × 10^−3^	Left
Frontal opercular cortex, frontal orbital cortex, frontal pole						
Middle temporal gyrus, angular gyrus and lateral occipital cortex	−58	−44	0	3.80	7.29 × 10^−5^	Left
Middle temporal gyrus and angular gyrus	52	−64	24	3.87	5.34 × 10^−5^	Right
Lateral occipital cortex	24	−82	−26	3.38	3.62 × 10^−4^	Right
Frontal pole	−10	60	20	3.81	6.92 × 10^−5^	Bilateral
Cingulate gyrus	0	−48	26	3.64	1.38 × 10^−4^	Bilateral
