# Supplementary material for: Auditory sequence processing reveals evolutionarily conserved regions of frontal cortex in macaques and humans
Source: Nat Commun. 2015 Nov 17;6:8901. doi: 10.1038/ncomms9901 (PMC4660034; doi:10.1038/ncomms9901)
Supplement: Supplementary Information — Supplementary Figures 1-8, Supplementary Notes 1-8 and Supplementary References [file ncomms9901-s1.pdf]

1. Supplementary Figures

I. Language specific and domain general processes in humans and macaques

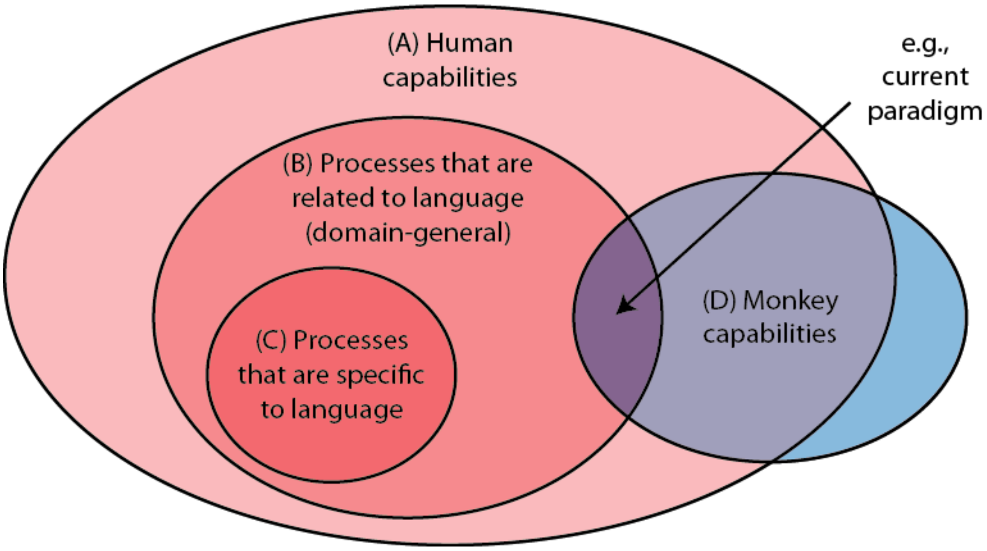

Supplementary Figure 1. Schematic represenation of human and macaque capabilities, highlighting the space that the current study explores. (A) Within the range of human capabilities are abilities related to langauge processes (B). Some of these are unique to human language ('language specific') and as such cannot be studied in nonhuman animals (C). A subset of language-related abilities that are not language specific (i.e., cognitive domain-general processes) support language and other aspects of cognition (shown as the area within B that excludes C). Monkeys possess their own set of cognitive capabilities (D), none of which would overlap with language-specific processes, but might overlap with domain-general processes that are evolutionarily conserved (overlapping purple area). Sequence processing and statistical learning paradigms, like the one used here, are linked to human language-related processing by studies of human adults, children and infants, and are known to engage aspects of the language network, such as regions of ventral frontal cortex. Nonhuman primates are also capable of learning some of these sequencing operations.

II. Comparable patterns of behavioural results in macaques and humans.

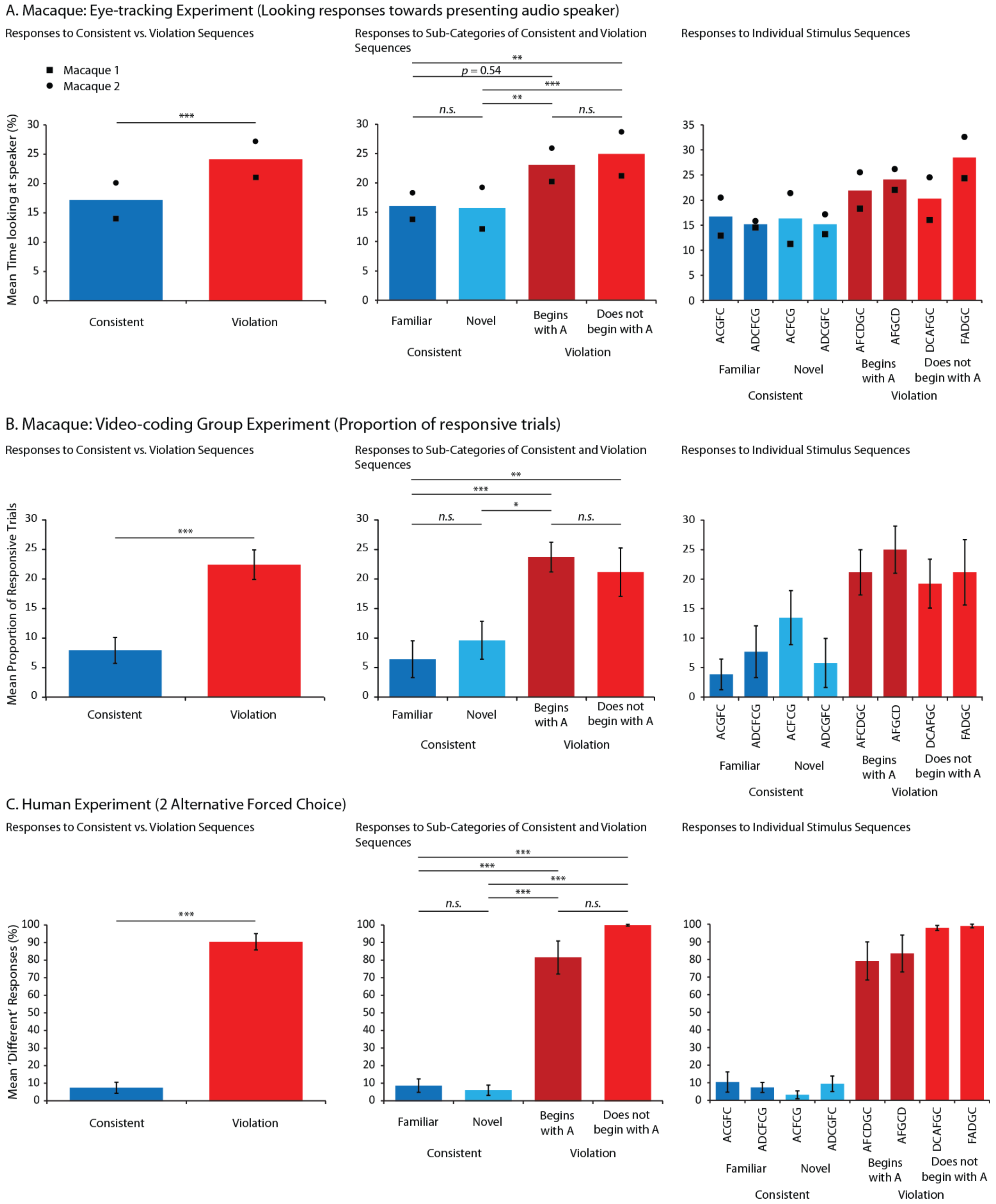

Supplementary Figure 2. Comparable patterns of behavioural results in macaques and humans. Beyond the categorical difference between 'consistent' and 'violation' sequences (manuscript Fig. 1), our experimental design allowed further subdivision of the behavioural results. Here we perform additional analysis of macaque and human data to determine whether the pattern of responses is comparable between species (see Supplementary Note IX). (A) Mean proportion of eye-tracking trials during which two of the Rhesus macaques (M1

and M2) that were scanned made looking responses to the consistent and violation conditions, including additional subdivisions of the testing sequences (middle column) and responses to individual testing sequences (right column). Each animal's individual looking responses are also shown (M1= square; M2 = circle). (B) Mean (±SEM) proportion of trials on which 13 macaques looked to the testing sequences in a video coding experiment. For more information see [1]. (C) Mean (±SEM) proportion of trials in which 12 humans responded that the testing sequences are 'different' relative to those heard during exposure. *n.s.* = not significant; *=p<0.05; **=p<0.01; ***=p<0.001.

III. Statistical properties of the sequences predict behavioural responses in humans and monkeys

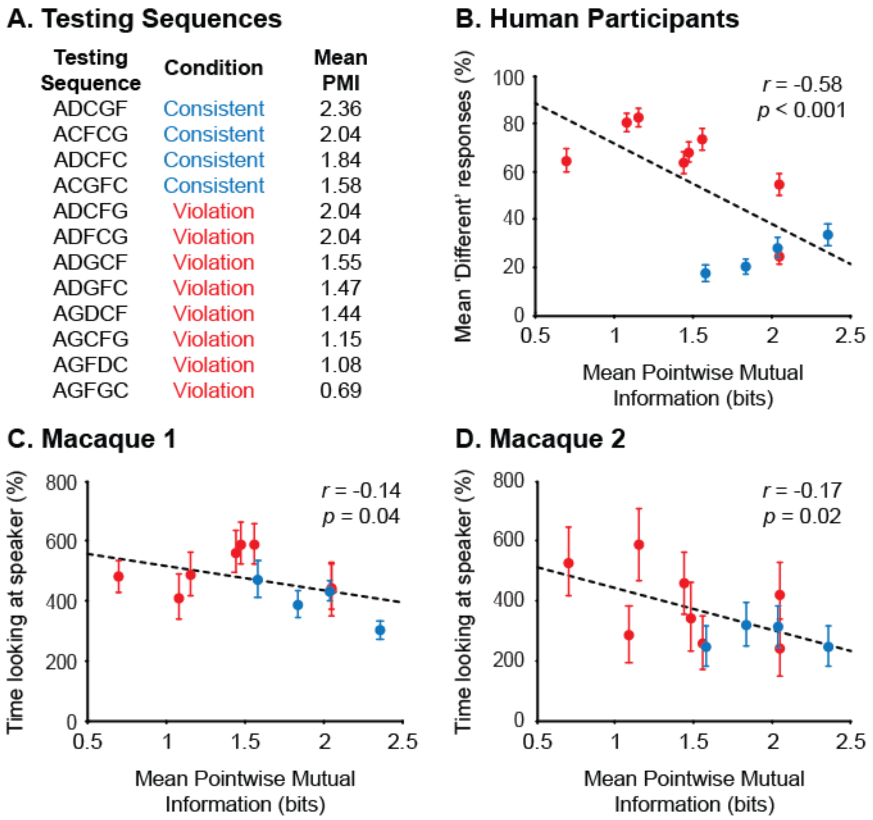

Supplementary Figure 3. Statistical properties of the sequences predict responses in humans and monkeys. To investigate whether macaque and/or human responses vary as a function of the predictability of the testing sequences, we conducted an additional behavioural experiment in both species (see Supplementary Note X). In this experiment the transitions in the testing sequences were more varied in terms of their predictability (as measured by pointwise mutual information, PMI). (A) The 4 consistent (blue) and 8 violation testing sequences (red) varied considerably in their average PMI. (B) The mean (±SEM) proportion of human participants' responses in a two-alternative forced-choice experiment showed a strong negative correlation with the average PMI of the sequences. (C-D) The mean (±SEM) proportion of each eye-tracking trial during which two macaques looked at the audio speaker presenting the test sequences also show significant negative correlations with the average PMI of the test sequences. These results demonstrate that both monkey and human responses are associated with the statistical properties of the sequences which are established during exposure to the consistent sequences prior to testing.

IV. Brain regions sensitive to sequence ordering violations in Macaque 3 displayed on a sagittal slice.

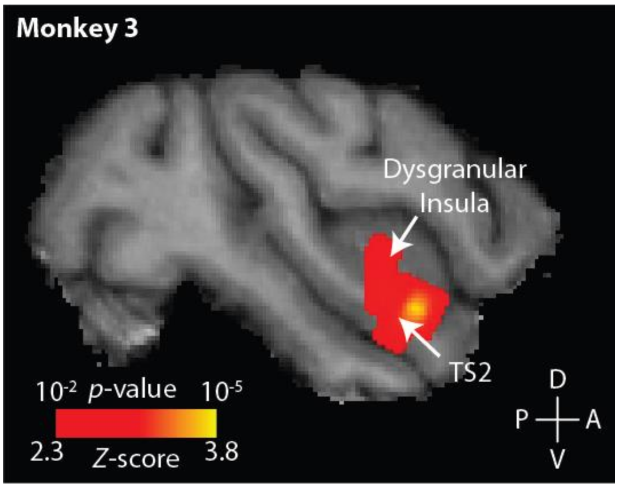

Supplementary Figure 4: Brain regions sensitive to sequence ordering violations in Macaque 3 displayed on a sagittal slice. Activation in response to sequence violations (contrast: Violation vs. Consistent) showing the involvement of the dysgranular insula in the frontal operculum more clearly than the surface rendered maps in manuscript Fig. 2. Moreover, the ROI analyses complement this observation and demonstrate significant involvement of the vFOC, which includes the insula and frontal operculum in all three of the animals (see manuscript Fig. 3).

V. Macaque control experiment 1: Exposure to randomly sequenced nonsense words

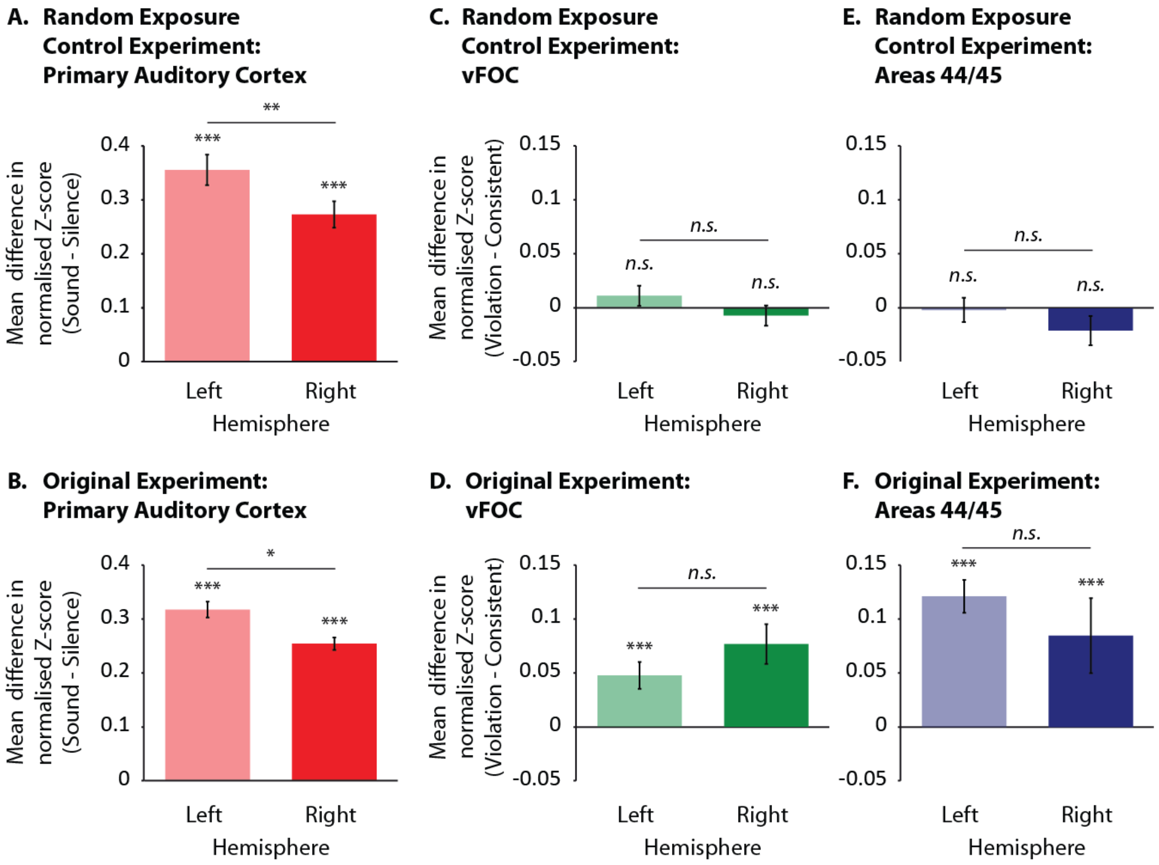

Supplementary Figure 5. Macaque control experiment 1: exposure to randomly sequenced nonsense words. We conducted an additional experiment in Macaque 1 to rule out the possibility that the ventral frontal cortex response reported in the manuscript (in the vFOC and/or Areas 44/45; manuscript Fig. 2-3) could be attributed to perceptual differences between the violation and consistent testing sequences, rather than depending on prior exposure to representative consistent sequences (see Supplementary Note. XI). This experiment was conducted identically to the original experiment, except that the monkey was exposed to randomly generated exposure sequences that provided no cues regarding what constitutes a 'consistent' or 'violation' sequence. (A-B) ROI analyses of activation in primary auditory cortex (area A1) in response to test sequences (including both consistent and violation sequences) relative to a silent baseline period. In both the control experiment (A) and the main experiment (B) comparable activation is observed to sounds relative to silence (control experiment, left hemisphere: t_{262}=21.30, p<0.001; right: t_{262}=21.931, p<0.001; two-sample t-test between hemispheres: t_{524}=3.349, p=0.001; Main experiment, left: t_{158}=12.590, p<0.001; right: t_{1588}=11.148, p<0.001; two-sample t-test between hemispheres in AC: t_{316}=2.205, p=0.028). This suggests that the fMRI data collected between experiments is well matched in its auditory fMRI response. (C-F) ROI analyses identical to those conducted in the main experiment were performed on the control data to assess whether differential activation might be observed in key ventral frontal regions following exposure to sequences containing no meaningful structure. In the control experiment no significant increase in activation to violation sequences relative to consistent sequences was observed in either vFOC or Areas 44/45 (C and E; vFOC, Left: t_{625}=1.198, p=0.462; Right: t_{642}=-0.749, p=0.908;

two-sample *t*-test between hemispheres in vFOC: t_{1267}=1.375, p=0.169. Areas 44/45, Left: t_{252}=-0.146, p=1.0; Right: t_{273}=-1.521, p=0.258; two-sample *t*-test between hemispheres in Areas 44/45: t_{525}=1.070, p=0.281). For comparison, the ROI analyses from the main experiment are also presented, showing significant responses to the violation vs. consistent sequences after exposure to representative sequences following the legal ordering relationships (D and F; Left vFOC: t_{366}=3.827, p<0.001; right vFOC: t_{404}=4.155, p<0.001. Two sample *t*-tests, between hemispheres in vFOC: t_{770}=1.279, p=0.201. Left Areas 44/45: t_{192}=8.000, p<0.001; right Areas 44/45: t_{181}=2.438, p=0.032; two sample *t*-tests, between hemispheres in Areas 44/45: t_{373}=0.977, p=0.329). Symbols: *n.s.* = not significant; *=p<0.05; **=p<0.01; ***=p<0.001.

VI. Macaque control experiment 2: fMRI activation as a function of the number of violations in the sequences

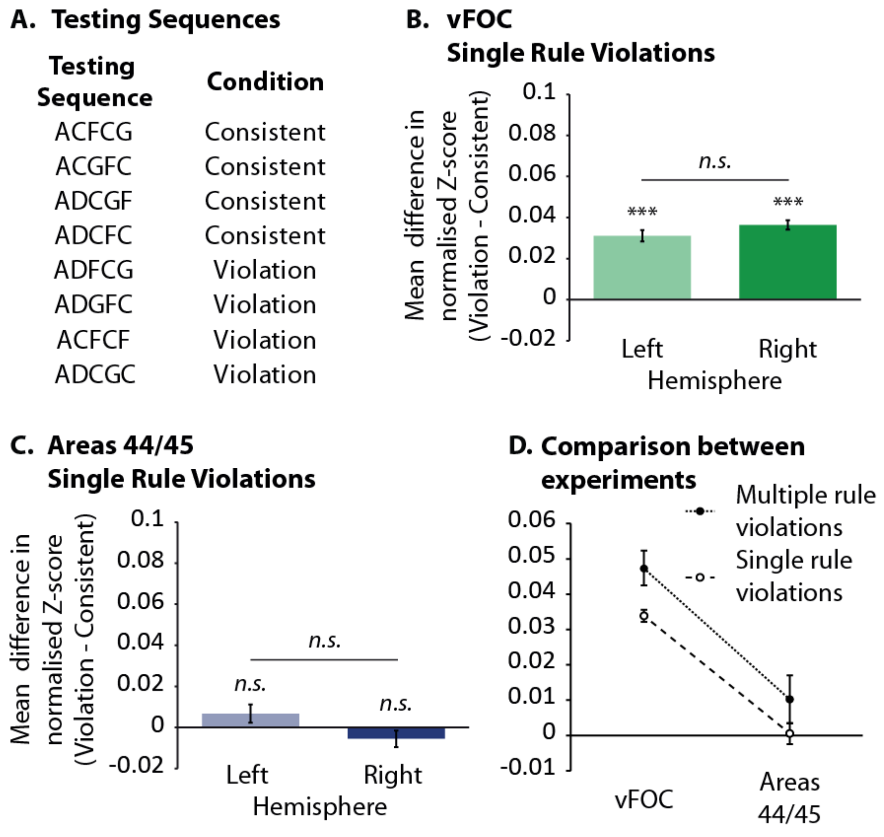

Supplementary Figure 6. Macaque control experiment 2: fMRI activation as a function of the number of violations in the sequences. We conducted a second control experiment using testing sequences which contained more subtle ordering violations than those in the main manuscript (see Supplementary Note XII). (A) Testing sequences used in this experiment. Each violation sequence contains only a single violation. (B) The planned ROI analyses show significant activation to the violation vs consistent contrast in the vFOC in both hemispheres: Left, t_{658}=11.327, p<0.001; Right, t_{702}, p<0.001, between hemispheres: t_{1360}=1.516, p=0.130. (C) No significant effect was observed for Areas 44/45: Left, t_{276}=1.513, p=0.262; Right, t_{282}=-1.371, p=0.342, between hemispheres: t_{558}=2.044, p=0.130. (D) An RM-ANOVA with the factors Condition (Violation and Consistent), ROI (vFOC and Areas 44/45) and Experiment (single violation experiment, and the ‘multiple rule violation’ main experiment presented in the manuscript) was performed to compare the patterns of activation observed between experiments. There was a significant main effect of Condition ($F_{1,3840}$=191.462, p<0.001), demonstrating that these key ventral frontal ROIs respond more strongly to violation sequences than consistent sequences. There was also a strong interaction between Condition and Experiment ($F_{1,3840}$=55.048, p<0.001), showing that the BOLD response to the violations was stronger in the experiment containing a higher number of such violations. There was also an interaction of Condition and ROI ($F_{1,3840}$=5.856, p=0.016), demonstrating that the vFOC was more involved in sequence processing than Areas 44/45, and a further interaction between Condition, ROI and Experiment was seen ($F_{1,3840}$=20.505, p<0.001), showing that stronger response in vFOC relative to Areas 44/45 was more pronounced in the single violation experiment.

VII. ROI analyses in temoporo-parietal Area 7 in the macaques and Area 39 in the human participants.

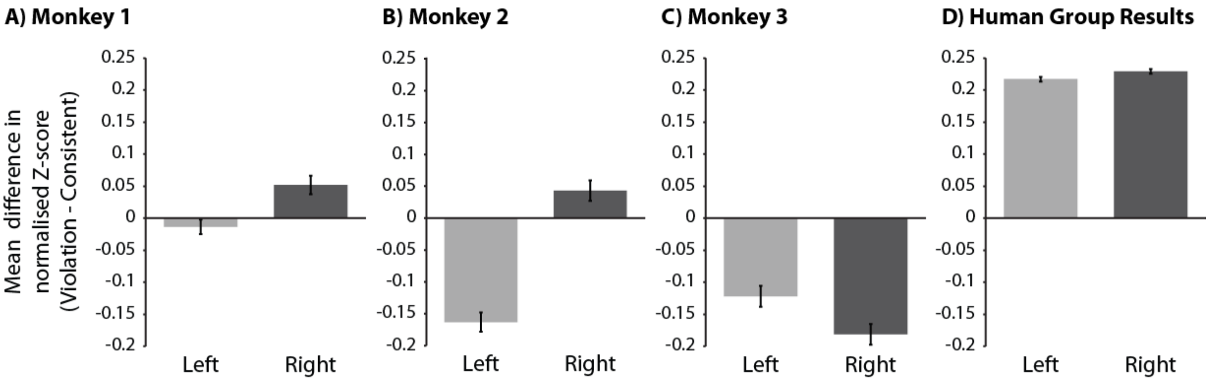

Supplementary Figure 7: ROI analyses in Area 7 in the macaques and Area 39 in the human participants. The whole brain GLM analyses revealed considerable activation in the inferior parietal lobule in both species. We anatomically defined regions of interest for these areas and assessed activation in response to violation vs consistent sequences in each of the macaques and the human participants. This analysis identified sensitivity to violations of the sequence ordering relationships in the right hemisphere Area 7 ROI in monkeys 1 and 2, consistent with the whole brain results. No such activation was observed in monkey 3, or in the left hemisphere of any of the monkeys. Strong bilateral activation was observed in human BA39, in line with the robust activation seen at the group level. These analyses support our conclusion that while activation in vFOC appears to be highly consistent between the individuals and species, other regions show much more variable responses to violations of the sequence ordering relationships.

VIII. Human fixation task during fMRI sequence processing

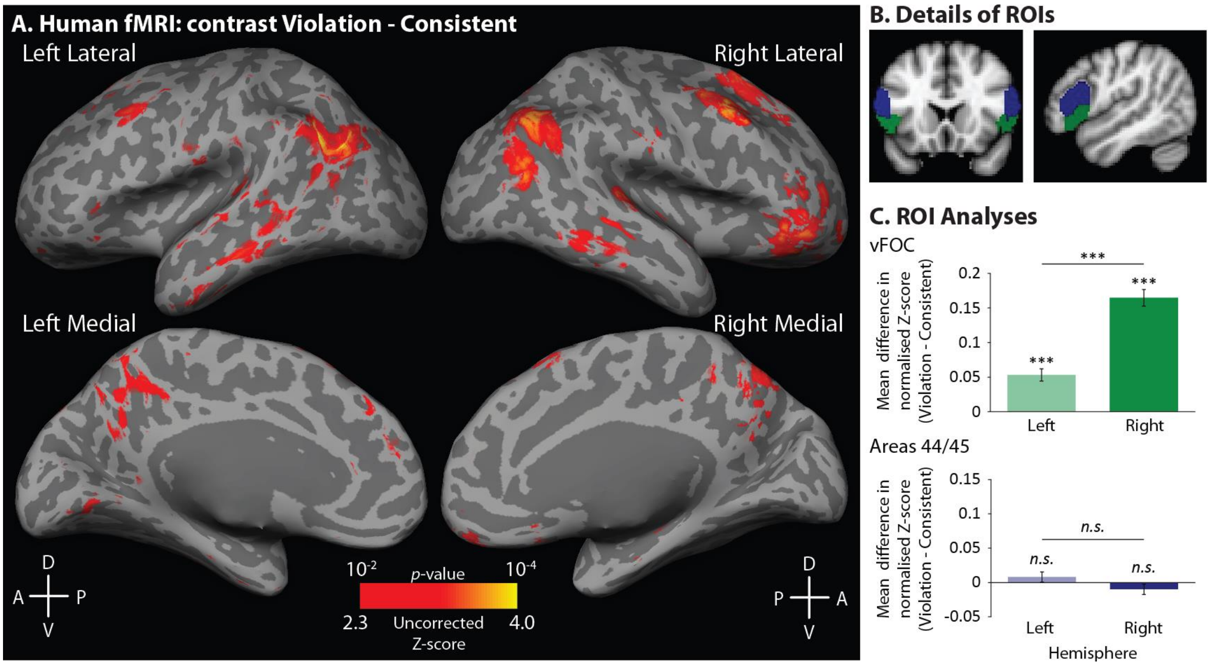

Supplementary Figure 8. Human fixation task during fMRI associated with sequence processing. We conducted a further fMRI experiment in which 4 human participants were scanned using the same visual fixation task as we used in the macaques (passively listening to the testing sequences during scanning, see Supplementary Note XIII). (A) Human group statistical parametric map of sensitivity to sequence violations (contrast: violation vs. consistent). Format as in manuscript Fig. 4. Results are displayed on rendered medial and lateral surface representations (light grey: gyri; dark grey: sulci) transformed to the MNI standard brain and using human anatomical atlases (Methods). (B) vFOC (green) and Areas 44/45 (blue) ROIs. (C) Normalised mean ROI voxel response differences (violation vs. consistent) in the fMRI signal for vFOC and Areas 44/45 in both left and right hemispheres of the human brain. Additional analyses to investigate activation in these two ROIs independently showed that the vFOC region was strongly sensitive to sequence violations bilaterally, while the Areas 44/45 region showed no significant sensitivity to violation sequences. One-sample *t*-tests with Bonferroni correction, in left vFOC: t_{961}=5.918, $p<0.001$; right vFOC: t_{976}=13.653, $p<0.001$; two-sample *t*-test between hemispheres in vFOC: t_{1937}=7.399, $p<0.001$. One-sample *t*-tests with Bonferroni correction, in left Areas 44/45: t_{730}=1.074, p=0.564; right Areas 44/45: t_{654}=-1.327, p=0.370; two-sample *t*-test between hemispheres in Areas 44/45: t_{1384}=1.695, p=0.09. Symbols: *n.s.* = not significant; ***=$p<0.001$.

2. Supplementary Notes

I. Sequence processing paradigms and statistical learning. Statistical learning is a broad term that can encompass many different forms of learning [2]. In our case, the form of statistical learning that the behavioural results in both species support is the implicit learning of the relationships between elements in a sequence as a function of the probabilities with which they occur in the exposure corpus [2,3]. This type of process, has been argued to represent a critical initial step in sentence processing in the human brain [4]. Furthermore, this form of sequence processing has been suggested as a potential language precursor process and could be studied in extant nonhuman primates [5].

The sequences used here have considerable variability in their composition and length, and emulate some of the properties of language and bird song. For instance, sentences also vary in length and composition and often contain dependencies between words whereby the presence of one word predicts others. These relationships can be deterministic (e.g. the presence of an article like 'the' or 'a' requires the presence of a noun later in the sentence) or probabilistic (e.g. the verbs 'think' or 'agree' can both optionally be followed be the complementizer 'that', but differ in their tendency to do so) [6]. Similar probabilistic relationships govern the combinations of phonemes which occur within and between words in a language, a property which even young children are sensitive to [7]. These low-level statistical properties are likely required to bootstrap the learning of higher-level structural features of syntax [8]. Probabilistic sequencing abilities can also be seen in animal communication. Using bird song as an example [9,10], a motif 'A' might precede either motif 'B' or 'C' with some predictability, whereby both A to B and A to C are legal song transitions. However, the transition of C to B might be illegal and could elicit a longer orienting response in the listener. In previous behavioural studies we showed that Rhesus macaques and humans are comparably sensitive to sequences that violate adjacent ordering relationships throughout the sequences [1,11].

II. Analysis of 'violation' vs. 'consistent' results in the artificial grammar and sequence processing neuroimaging literature. After exposure to representative consistent sequences, sequence processing paradigms test whether participants respond differently to consistent relative violation sequences, to determine whether some form of learning has occurred. The way in which the violation sequences are designed provides information on the types of violations of the sequence ordering relationships that the participant is sensitive to (e.g., Supplementary Fig. 2-3; also see [1]. Similarly, the fMRI contrast between violation and consistent sequences asks which brain regions/voxels are sensitive to ordering violations (as is common in most human fMRI studies of AGL; e.g. [4,12-15]; although see [16]). This contrast would disregard brain areas involved in processing the acoustics of the sequences, which should be comparable across the consistent and violation sequences if acoustic differences are balanced between the sequences, as they were for this study. Thus, fMRI responses to this contrast would identify brain areas sensitive to illegal or unexpected transitions between sequence elements based on the ordering relationships previously learned during the exposure phase of the experiment. Beyond representing a neuroimaging analogue of traditional behavioural experiments, this contrast has also revealed activation in the same brain areas that are involved in processing key aspects of language [4,12-15]. Therefore, in humans these brain areas are not only involved in detecting violations of sequence ordering

relationships, but are also critically involved in processing natural language, making such sequence processing experiments a potentially useful way to investigate the function of these key ventral frontal brain areas.

III. Summary of fMRI lateralisation results in macaques and humans. We observe very few significant lateralisation effects in this study. Although the macaque results in manuscript Fig. 2-3 appear to demonstrate more activation in the right than the left hemisphere, statistical analyses of the activation in the key ROIs provide little evidence for significant lateralisation effects. Specifically, we observe no significant lateralisation in the vFOC, (1) in the human explicit experiment (manuscript Fig. 4), (2) in the majority of monkeys in manuscript Fig. 2, (3) even the one monkey that shows significant right lateralisation in manuscript Fig. 3B shows no significant lateralisation in the 'single' violation experiment (Supplementary Fig. 6). The one clear example of right lateralisation is in the implicit human fMRI experiment (Supplementary Fig. 8), so tentatively task differences might influence the lateralisation of effects. However, further experiments would need to be conducted with better matched numbers of human participants performing different tasks. Therefore, we cannot conclude that the brain areas involved in these tasks are significantly lateralised. However, we also cannot claim that the network associated with this type of sequence processing task is bilaterally distributed, since this interpretation depends on a null statistical difference between the hemispheres, which can occur for a number of reasons. Our results provide novel evidence regarding the brain areas associated with this form of sequence processes in humans and nonhuman primates, which overall do not seem to be significantly left or right lateralised.

IV. Variation of macaque looking responses to violation vs. consistent sequences. Although the macaques were engaged in a fixation task to limit eye movement effects, we wondered whether the variability in eye movements during sequence presentation (possibly reflecting salient, attention-getting aspects of the stimuli or the experimental environment) was associated with the experimental sequences. In the two macaques for which eye-tracking data were recorded (M2, M3), we compared the amount of eye movements made during the presentation of the consistent and violation testing sequences. However, neither monkey showed any significant difference in the variability of eye movements in response to the violation sequences (M2: t_7=0.63, p=0.55; M3: t_4=-0.33, p=0.75). Thus it is unlikely that eye movements and the animals' eye looking responses during fMRI sequence testing significantly impacted on the results obtained.

V. Additional cross-species analyses separated by ROI. The combined cross-species RM-ANOVA results for the vFOC and Areas 44/45 ROIs (in the manuscript) were supported by two separate RM-ANOVAs, each including only a single ROI. Both of these showed a main effect of Condition (vFOC: $F_{1,5311}$=408.064, p<0.001; Areas 44/45: $F_{1,2732}$=14.651, p<0.001) demonstrating sensitivity in these regions across the species. In both analyses, there was also an interaction of Condition and Species. However, this effect occurred in opposite directions suggesting that vFOC activation is relatively stronger in humans than monkeys ($F_{1,5311}$=67.51, p<0.001), while monkeys show more activation in Areas 44/45, relative to human

participants ($F_{1,2732}$=8.22, p=0.004). Together these results demonstrate that increased activation is observed in ventral frontal opercular cortex in both species.

VI. Relationship and differences to paradigms with oddball sounds. The current paradigm and the results of this study are markedly different from other types of commonly used sequence processing tasks, based on the complexity of the ordering relationships in a sequence, the timing of the effects (whether the learning occurs between trials separated by a few seconds or following a long period of exposure), the length of the sequences, and the variability in transitional probabilities between elements in a sequence. The properties of this sequence processing paradigm have been compared within a quantitative parameter space to features of other related tasks that have been used to study nonhuman animals [17,18], also see [19]. Because of the variability in its transitional properties and generally longer and more variable sequences than other paradigms, it can be characterised as moderate in complexity. It is also a simpler version of the original Reber artificial grammar [20-23], which at least Rhesus macaques are able to learn [1,11].

By comparison, traditional oddball paradigms present a stream of 'standard' stimuli (e.g., tones of a certain pitch), infrequently interrupted by an unexpected, attention-getting, 'deviant' stimulus (e.g., a tone of a different pitch) [24,25]. Some studies have used more complex oddball paradigms, in which the 'standard' stimulus might be a short series of tones in a fixed pattern (for example AAB, where A and B represent tones of different pitches), and the oddball, deviant stimulus might be a different series of tones (e.g., AAA) [24,25]. In both these cases, detecting the deviant stimuli involves noticing a perceptual difference in the stream (either a change in the pitch of the tones or the sequence of tones). By contrast, the current paradigm contains no 'oddball' sounds in any of our sequences: the same 5 nonsense word elements appear in both the violation and consistent sequences. Therefore none of the individual elements represent 'deviants' in the same way as in oddball tasks. In addition, our control experiment demonstrates that the violation testing sequences fail to elicit any differential activation in relation to the consistent sequences in the vFOC when the animal was exposed to sequences containing random transitions between the 5 elements (Supplementary Fig. 5). Furthermore, the graded responses elicited by sequences containing larger numbers of illegal, violation transitions (Supplementary Fig. 3 & 6), suggest that the responses we observe cannot be interpreted as a binary categorical 'deviant' vs 'standard' response.

Moreover, these different paradigms appear to be supported by fundamentally different brain areas. Auditory oddball, deviant-detection tasks (e.g., with attention-getting, deviant stimuli) do not tend to recruit the ventral frontal regions implicated in the hypothesis we were testing here [4], and instead tend to engage a more general deviance detection network [26] belonging to the dorsal, rather than the ventral, processing stream [4,27,28]. For instance, simple oddball paradigms, in which the standard and deviant stimuli are tones of different pitches, appear to primarily engage auditory cortex. More complex oddball paradigms, such as those in which the participant is required to notice the unexpected sequence (AAA) within the stream of repeated presentations of the standard sequence (AAB), produce activation in a distributed dorsal parietal network [24,25]. These tasks do not appear to strongly engage the ventral frontal cortex [24-26], unlike the current sequence order learning paradigm. Interestingly, oddball paradigms using more language-like stimuli in humans have revealed a

syntactic mismatch negativity (sMMN) MEG response, localised to the inferior frontal cortex [29]. However, the inferior frontal sources are only observed when the oddball stimulus represents both a perceptual and a syntactic deviant.

Sequence processing operations vary considerably in complexity and appear to engage certain regions of frontal cortex and other brain areas depending on the demands imposed by the task and sequencing process. In other words, if deviance detection processes, or by other accounts prediction errors [30,31], are at play in these ventral frontal regions, these depend entirely upon the sequence processing demands imposed here. Any violation, deviance or prediction-error only obtains this status as a function of violating the ordering relationships that the participant encoded during the prior exposure phase. This is supported by the human and monkey behavioural sensitivities (see Supplementary Figs. 2-3 and [1,11]) and the two monkey fMRI control experiments (Supplementary Fig. 5-6), all of which show a dependence on the statistical properties of the sequencing relationships established by the regularities in the sequences that the participant heard during exposure.

VII. Other potential differences between the species. The humans and monkeys were scanned with different scanners, and any differences between how the two species were tested need to be considered when interpreting the results. For this study, the relatively smaller macaques were scanned in a vertical scanner at a higher magnetic field strength (4.7 Tesla) while the humans were scanned lying horizontally at 3 Tesla. Nonetheless, every effort was taken to ensure that the scanning sequences and parameters were comparable between the species, while accommodating the different sized brains of the two species (see Methods). It is striking that amidst any such differences there is considerable consistency in the vFOC activity response to violation sequences between the humans and macaques. Moreover, behavioural strategies across the species seemed to be largely comparable (Supplementary Fig. 2-3) and task differences cannot easily explain the reported effects (compare manuscript Fig. 4 with Supplementary Fig 8 in humans, and also to manuscript Fig. 2-3 in macaques).

VIII. Assessing potential gender differences in the human participants. To investigate whether there were any gender differences between our human participants (6 male and 6 female) we contrasted activation observed in response to the violation vs consistent contrast in males and females. No significant activation between males and females or vice versa survived ($p<0.05$ cluster corrected) anywhere in the brain including in the key ROIs. Even using a more liberal statistical threshold ($p<0.001$ uncorrected), no differences between the genders were observed.

IX. Comparable patterns of behavioural results in macaques and humans (associated with Supplementary Fig. 2). We conducted further analyses of the behavioural data presented in the manuscript to determine whether humans and monkeys produced different patterns of responses, which would suggest that they used different learning strategies or were sensitive to different properties of the sequences. These additional results show that as

far as we can measure the pattern of results is largely comparable across the species (see Supplementary Fig. 2).

Testing for simple learning strategies in macaques and humans. Beyond the categorical difference between 'consistent' and 'violation' sequences (manuscript Fig. 1), our experimental design allowed further subdivision of the behavioural results, which previously ruled out certain types of simple learning strategies in macaques, see: [1]. Here we conduct a similar analysis of the human data to determine whether the pattern of results is comparable to those in macaques. To ensure that responses could not be attributed to rote memorisation or the familiarity of the sequences, the consistent testing sequences in this experiment consisted of two types: 'novel' and 'familiar'. The familiar sequences had been heard during the exposure phase of the experiment. By contrast, the novel sequences had not been heard by the humans or macaques prior to the testing sessions. Additionally we compared violation sequences that violate the sequencing relationships from the first element (sequences that 'do not begin with A') and those which, just like the consistent sequences, 'begin with A'. Response differences between 'familiar' and 'novel' sequences would suggest a reliance on the familiarity of the consistent sequences. Similarly, stronger responses to violation sequences that 'do not begin with A' relative to those that 'begin with A' could be attributed to only noticing simple initial violations. However, differences in responses between 'novel' consistent sequences and violation sequences that 'begin with A' would support the use of a strategy that goes beyond familiarity and noticing simple initial violations. Supplementary Fig. 2 (A-C, middle panels) demonstrates that both macaques and humans showed a significant difference in responses to sequences that 'begin with A' compared to 'novel' sequences, but no significant differences between 'familiar' and 'novel' sequences or those that 'begin with A' relative to those that 'do not begin with A'. These results suggest that both species used comparable, non-trivial strategies.

Macaque eye-tracking results: In the macaques, we analysed eye-tracking data from two of the animals that were scanned with fMRI, as well as the video-coding results obtained from a larger group of macaques (for more details see manuscript Methods and [1]). The eye-tracking data were analysed with an RM-ANOVA analysis including the factor Condition (with the levels 'familiar', 'novel', 'begin with A' and 'do not begin with A') and the between-subjects factor of Monkey (Supplementary Fig. 2A, middle panel). The results show a strong main effect of Condition ($F_{3,144}$=16.426, p<0.001) and no interaction between Condition and Monkey ($F_{3,144=}$0.202, p=0.655; Supplementary Fig. 2A, middle panel). Bonferroni corrected post-hot comparisons revealed no difference between 'familiar' and 'novel' consistent sequences or violation sequences that 'begin with A' compared to those that 'do not begin with A' (p=1.0 in both cases). Significant differences were observed between 'familiar' vs 'do not begin with A' (p=0.01); 'novel' vs 'begin with A' (p=0.002); 'novel' vs 'do not begin with A' (p=0.001) and the fourth comparison showed a statistical trend for 'familiar' vs 'begin with A' (p=0.054). These results suggest that the macaques' responses cannot easily be attributed to simple strategies such as memorisation of the exposure sequences or simply responding to those sequences containing violations in the initial element position.

Macaque video-coding results: The eye-tracking results from the two macaques that were scanned in this experiment are comparable to those from a previous video-coding experiment in which 13 Rhesus macaques were tested with the same sequences [1]. Here, after exposing the monkeys to representative consistent sequences, the monkeys were presented with the

testing sequences from a concealed audio speaker. Orienting responses to the speaker were videotaped and blind coded by three independent raters (for more details, see [1]). The macaques responded to a significantly higher proportion of the violation sequences than the consistent sequences (paired samples t-test, t_{12}=7.898, p<0.001; Supplementary Fig. 2B, left panel) and an RM-ANOVA revealed a strong main effect of sequence condition ($F_{3,36}$=9.146, p<0.001). As in the previous experiment, Bonferroni corrected post-hoc tests showed no differences within the consistent and violation conditions but revealed significant differences between a number of the conditions, including between 'novel' sequences and those that 'begin with A' (see Supplementary Fig. 2B, middle panel).

Human results: The human data reported in the main manuscript were further analysed using an RM-ANOVA with the Condition factor (levels: 'familiar', 'novel', 'begin with A' and 'do not begin with A'). This analysis revealed a strong main effect of Condition ($F_{3,33}$=91.277, p<0.001; Supplementary Fig. 2C, middle panel). Furthermore, Bonferroni corrected post-hoc tests revealed no differences between the 'familiar' and 'novel' consistent sequences (p=1.0) or the violation sequences that 'begin with A' relative to those that 'do not begin with A' (p=0.5). All four contrasts between the consistent and violation conditions were highly significant (p<0.001). These results suggest that the human participants' responses could not be attributed to simple strategies such as memorisation of the exposure sequences or simply responding to those sequences containing violations in the initial element position. These results in human participants (Supplementary Fig. 2C) closely mirror those obtained in the macaques (Supplementary Fig. 2A-B), suggesting that both species use similar strategies and demonstrate that the responses of neither species can be attributed to simple learning strategies.

Additionally, we report the behavioural results in the macaques and humans to each of the testing sequences individually (Supplementary Fig. 2A-C, right panels). Here the results to the 8 sequences are too variable and underpowered to statistically analyse in the same way. Nonetheless, the pattern of monkey and human results is at least qualitatively comparable (showing a notable difference between the consistent and violation sequences, relative to much smaller differences within these conditions). While these data in Rhesus macaques and human participants were measured differently (humans with button presses; macaques with looking responses), the results nonetheless demonstrate that both species show very similar patterns of responses and neither species relies on certain simple learning strategies (unlike the results previously obtained from marmoset monkeys [1]).

X. Statistical properties of the sequences predict behavioural responses in humans and monkeys (associated with Supplementary Fig. 3). All of the violation sequences used in the experiments in this study contained multiple violations of the sequencing relationships (see [1] for more details). These violations represent illegal transitions between elements, transitions which never appear in the consistent sequences. The statistical likelihood of any transition between two nonsense word elements can be calculated from the frequency with which that transition occurs in the exposure phase of the experiment. For example, Pointwise Mutual Information (PMI), which reflects the association between two nonsense word elements, can be calculated for each transition within the exposure sequences. An illegal transition between pairs of elements can never occur in the exposure sequences, and therefore has a PMI of 0. The mean PMI for all of the transitions within a sequence represents an

average measure of how predictable or expected the transitions within that sequence might be, with high values representing more highly predictable, consistent sequences, and lower values corresponding to sequences with more unexpected or illegal transitions. In this initial experiment using a sequence learning paradigm and fMRI in macaques and humans, we used violation sequences containing multiple violations. Therefore, there were clear categorical differences between the average PMI of the consistent and violation sequences (the mean PMIs in the consistent sequences were 1.9, 2.1, 1.9 and 2.2 bits compared to 0.50, 0.74, 0.62 and 0.44 bits in the violation sequences; see Table 1 in [1] for more details).

To investigate whether macaque and/or human responses vary as a function of the predictability of the testing sequences, we conducted an additional behavioural experiment in both species using testing sequences containing a wider range of PMIs (Supplementary. Fig 3). This allows us to compare behavioural responses to sequences containing a range of different PMIs, including sequences with very low average PMIs as well as violation sequences with PMIs more comparable to the 4 consistent sequences used (Supplementary Fig. 3A). As with the original experiment reported in the manuscript (manuscript Fig. 1) and above (Supplementary Fig. 2) an eye-tracking experiment was conducted in 2 Rhesus macaques (16 testing sessions each) and a forced-choice experiment in 33 human participants. Identical stimuli were used in both experiments.

The results of the macaque eye-tracking experiment demonstrate that both monkeys' looking responses in the direction of the presenting audio speaker were negatively correlated with the average PMIs of the sequences (M1: r=-0.143, p=0.040; M2: r=-0.169, p=0.019; Supplementary Fig. 3C-D). Thus the monkeys' looking responses scaled with the level of predictability in the sequences, with stronger looks to less predictable sequences. In the human experiment, participants' 'different' responses (i.e., responses that the testing sequences were different from the representative consistent sequences heard during exposure) also showed a negative correlation with the average PMI of the test sequence (r=-0.583, p<0.001). This result demonstrates that more unpredictable test sequences, with lower average PMIs, are more likely to be classified as violation sequences (Supplementary Fig. 3B).

These results show that both humans' and monkeys' responses to the testing sequences are associated with the statistical properties of the sequences. In this experiment and the original behavioural experiment (manuscript Fig. 1, Supplementary Fig. 2), the macaques and humans were tested with different methods (humans with button presses, monkeys with eye-tracking). Such differences, alongside any differences in sample sizes, may account for some aspects of the results such as the stronger correlation in humans (Supplementary Fig. 3B; compare with Supplementary Fig. 2C-D) and make it difficult to directly compare magnitude differences (Supplementary Fig. 2). Nonetheless, the general pattern of responses in macaques and humans across these experiments is strikingly similar (Supplementary Figs. 2-3) and is interesting in relation to the fMRI results obtained (see manuscript Figs. 2-4 and Supplementary Figs. 5-6). The combined results suggest that both species rely on similar behavioural strategies, which are supported by a notable level of correspondence in specific ventral frontal cortical brain regions.

XI. Macaque control experiment 1: Exposure to randomly sequenced nonsense words (associated with Supplementary Fig. 5). We conducted an additional experiment with Macaque 1 (Supplementary Fig. 4) which supports the following conclusion: The ventral frontal cortex response reported in the manuscript (in the vFOC and/or Areas 44/45; manuscript Fig. 2-3) depends on prior exposure to representative consistent sequences (manuscript Fig. 1A), rather than being attributable to some other difference between the violation and consistent testing sequences (which were acoustically well matched see: Methods in main paper and also [1]).

We generated 100 randomised exposure sequences with a flat transitional probability distribution, matched for length with the exposure sequences in the main experiment. In these sequences every possible transition between elements occurred approximately equally often, with the constraint that, as in the main experiment, no nonsense word element was ever repeated consecutively. Unlike the exposure sequences used in the main experiment, these sequences provide no cues regarding what constitutes a 'consistent' or 'violation' sequence. Macaque 1 was exposed to these random sequences for the same length of time as in the original experiment, before being scanned with the 'consistent' and 'violation' testing sequences from the original experiment. The only difference between the experiments was that in this control experiment, during the exposure phase prior to scanning, the participant heard random sequences rather than the consistent, exposure sequences. We predicted that if the effects reported for this animal in the original experiment were due to any (for example acoustical) differences between the 'consistent' and 'violation' testing sequences, then these effects should persist in the control experiment. However, if the activation observed in the ventral frontal cortex depends on the relationships between elements to which the animal had been exposed, then no differences should be evident in the ventral frontal cortex in response to 'violation' vs. 'consistent' sequences.

To ensure that the dataset obtained was sufficiently powered to the one reported in the manuscript, the same amount of fMRI data was entered into each analysis. We also confirmed that the 'sound vs. silence' response in auditory cortex (cortical field A1) was comparable between the two datasets (compare Supplementary Fig. 5A-B). Following exposure to the random exposure sequences no significant (p<0.05 cluster corrected) fMRI activity response to violation vs. consistent was observed anywhere in the whole brain results, unlike the brain-wide results in the original experiment for this animal (manuscript Fig. 2A). Even using a more liberal analysis (p<0.01, uncorrected), no voxels in the vFOC or Areas 44/45 ROIs were significantly activated. ROI analyses identical to those conducted in the main experiment revealed no significant differences in activation to violation and consistent sequences in either vFOC or Areas 44/45 (Supplementary Fig. 5C, E). The results of this control experiment provide evidence that the activation in the main experiment, particularly in vFOC, cannot be attributed to, for instance, acoustical differences between the 'violation' and 'consistent' testing sequences, and instead represent a response to violation transitions based on previous exposure to the consistent exposure sequences.

XII. Macaque control experiment 2: fMRI activation as a function of the number of violations in the sequences (associated with Supplementary Fig. 6). The experiments presented here and in the MS demonstrate that key regions in ventral frontal cortex respond to sequences containing violations to sequence ordering relationships. This predicts that the

activity response should scale with the number of violations present in the testing sequences. To test this prediction, and to examine in more detail the role of these brain areas in processing sequences, we conducted a further control experiment on Macaque 2 (Supplementary Fig. 6). This experiment was conducted identically to the main fMRI experiment (manuscript Fig. 2-3), except here we used testing sequences designed to contain more subtle sequence ordering violations. Each violation sequence contained only a single sequence order violation, so that most elements in these violation sequences are ‘consistent’ with the legal ordering relationships of the paradigm (manuscript Fig. 1A), except for a specific illegal transition between a pair of elements (Supplementary Fig. 6A).

No voxels survived cluster correction in response to the violation-consistent contrast, suggesting that any effects in this experiment are less robust than in the main experiment. However, the planned ROI analyses, conducted identically to the main experiment, showed a comparable pattern of results. Significant activation was observed in the vFOC but not in the Areas 44/45 ROI (Supplementary Fig. 6B-C). In comparison to the original experiment (which has multiple violations in each sequence), these results show a reduced response pattern in the ventral frontal cortex ROIs to more subtle single sequence violations (Supplementary Fig 6). This experiment, along with the original ‘multiple violations’ experiment, suggest that the vFOC (and possibly also the Areas 44/45) fMRI response is modulated by the extent to which the testing sequences deviate from those to which the animal was previously exposed.

XIII. Human fixation task during fMRI sequence processing (associated with Supplementary Fig. 8). The human participants in the main experiment (manuscript Fig. 4) were tested using a two-alternative forced-choice procedure (identifying ‘consistent’ vs. ‘violation’ sequences with button presses) similar to methods typically used in the literature (e.g., [14,15]). Implicit sequence processing and statistical learning tasks in humans have shown comparable results in ventral frontal cortex but often require many more participants and still rely on button press responses [12,32,33]. Nonetheless, to determine whether the key pattern of activation in the ventral frontal cortex is also observed if humans are scanned using the same visual fixation task as we used in the macaques (passively listening to the testing sequences during scanning), we scanned 4 human participants with the fixation task (Supplementary Fig. 8). Unsurprisingly, the activation responses from this experiment were weaker than those from the main experiment reported in the paper, which had more participants and used explicit testing. Nonetheless, we saw a remarkably comparable pattern of brain-wide responses as reported in the main experiment (involving $p<0.001$ uncorrected voxels present in the key areas noted in the manuscript, including the vFOC; see Supplementary Fig. 8A).

ROI analyses identical to those performed in the main experiment were conducted on these data (Supplementary Fig. 8B-C). As in both the main human (manuscript Fig. 4) and the macaque fMRI experiment (manuscript Figs. 2-3), a significant activation response to the violation sequences was observed in the vFOC (although here it was significantly right lateralised; see below). Furthermore, as reported in the manuscript, no significant activation was observed in response to violation sequences relative to consistent ones in Areas 44/45 (Supplementary Fig. 8). These additional results in combination with those reported in the manuscript (manuscript Figs. 2-5) suggest that the shared patterns of ventral frontal cortex

activation across the species elicited by violation sequences can be observed independent of the behavioural task being performed.

3. Supplementary References

1. Wilson B, Slater H, Kikuchi Y, Milne AE, Marslen-Wilson WD, et al. (2013) Auditory artificial grammar learning in macaque and marmoset monkeys. J Neurosci 33: 18825-18835.
2. Pothos EM (2007) Theories of artificial grammar learning. Psychological bulletin 133: 227.
3. Conway CM, Pisoni DB (2008) Neurocognitive basis of implicit learning of sequential structure and its relation to language processing. Annals of the New York Academy of Sciences 1145: 113-131.
4. Friederici AD (2011) The brain basis of language processing: from structure to function. Physiol Rev 91: 1357-1392.
5. Fitch WT, Friederici A (2012) Artificial grammar learning meets formal language theory: An overview. Philos Trans R Soc Lond B Biol Sci 367: 1933-1955.
6. Jaeger TF (2010) Redundancy and reduction: Speakers manage syntactic information density. Cognitive psychology 61: 23-62.
7. Jusczyk PW, Luce PA (1994) Infants′ sensitivity to phonotactic patterns in the native language. Journal of Memory and Language 33: 630-645.
8. Mintz TH (2003) Frequent frames as a cue for grammatical categories in child directed speech. Cognition 90: 91-117.
9. Berwick RC, Beckers GJ, Okanoya K, Bolhuis JJ (2012) A Bird's Eye View of Human Language Evolution. Front Evol Neurosci 4: 5.
10. Bolhuis JJ, Okanoya K, Scharff C (2010) Twitter evolution: converging mechanisms in birdsong and human speech. Nat Rev Neurosci 11: 747-759.
11. Wilson B, Smith K, Petkov CI (2015) Mixed-complexity artificial grammar learning in humans and macaque monkeys: evaluating learning strategies. European Journal of Neuroscience 41: 568-578.
12. Udden J, Ingvar M, Hagoort P, Petersson KM (2012) Implicit Acquisition of Grammars With Crossed and Nested Non-Adjacent Dependencies: Investigating the Push-Down Stack Model. Cogn Sci.
13. Petersson KM, Forkstam C, Ingvar M (2004) Artificial syntactic violations activate Broca's region. Cognitive Science 28: 383-407.
14. Bahlmann J, Schubotz RI, Friederici AD (2008) Hierarchical artificial grammar processing engages Broca's area. Neuroimage 42: 525-534.
15. Friederici AD, Bahlmann J, Heim S, Schubotz RI, Anwander A (2006) The brain differentiates human and non-human grammars: functional localization and structural connectivity. Proc Natl Acad Sci U S A 103: 2458-2463.
16. Skosnik PD, Mirza F, Gitelman DR, Parrish TB, Mesulam MM, et al. (2002) Neural correlates of artificial grammar learning. Neuroimage 17: 1306-1314.
17. Kikuchi Y, Attaheri A, Milne A, Wilson B, Petkov CI (2013) Cortical oscillations and spiking activity associated with Artificial Grammar Learning in the monkey auditory cortex. SfN presentation.
18. Petkov CI, Wilson B (2012) On the pursuit of the brain network for proto-syntactic learning in non-human primates: conceptual issues and neurobiological hypotheses. Philos Trans R Soc Lond B Biol Sci 367: 2077-2088.
19. Hurford JR (2012) The Origins of Grammar: Language in the Light of Evolution II: Oxford University Press.
20. Reber AS (1967) Implicit learning of artificial grammars. Journal of Verbal Learning and Verbal Behaviour 6: 855-863.

21. Folia V, Udden J, De Vries M, Forkstam C, Petersson KM (2010) Artificial language learning in adults and children. Language Learning 60: 188-220.
22. Petersson K-M, Folia V, Hagoort P (2012) What artificial grammar learning reveals about the neurobiology of syntax. Brain and language 120: 83-95.
23. Saffran J, Hauser M, Seibel R, Kapfhamer J, Tsao F, et al. (2008) Grammatical pattern learning by human infants and cotton-top tamarin monkeys. Cognition 107: 479-500.
24. Uhrig L, Dehaene S, Jarraya B (2014) A hierarchy of responses to auditory regularities in the macaque brain. J Neurosci 34: 1127-1132.
25. Bekinschtein TA, Dehaene S, Rohaut B, Tadel F, Cohen L, et al. (2009) Neural signature of the conscious processing of auditory regularities. Proceedings of the National Academy of Sciences 106: 1672-1677.
26. Sabri M, Liebenthal E, Waldron EJ, Medler DA, Binder JR (2006) Attentional modulation in the detection of irrelevant deviance: a simultaneous ERP/fMRI study. Journal of Cognitive Neuroscience 18: 689-700.
27. Bornkessel-Schlesewsky I, Schlesewsky M, Small SL, Rauschecker JP (2015) Neurobiological roots of language in primate audition: common computational properties. Trends Cogn Sci 19: 142-150.
28. Rauschecker JP (1998) Parallel processing in the auditory cortex of primates. Audiol Neurootol 3: 86-103.
29. Hanna J, Mejias S, Schelstraete M-A, Pulvermüller F, Shtyrov Y, et al. (2014) Early activation of Broca’s area in grammar processing as revealed by the syntactic mismatch negativity and distributed source analysis. Cognitive neuroscience 5: 66-76.
30. Rao RPN, Ballard DH (1999) Predictive coding in the visual cortex: a functional interpretation of some extra-classical receptive-field effects. Nature neuroscience 2: 79-87.
31. Friston K (2010) The free-energy principle: a unified brain theory? Nature Reviews Neuroscience 11: 127-138.
32. Petersson KM, Folia V, Hagoort P (2012) What artificial grammar learning reveals about the neurobiology of syntax. Brain Lang 120: 83-95.
33. Folia V, Petersson KM (2014) Implicit structured sequence learning: an fMRI study of the structural mere-exposure effect. Frontiers in Psychology 5: 1-13
